# A network pharmacology and molecular docking investigation on the mechanisms of *Shanyaotianhua* decoction (STT) as a therapy for psoriasis

**DOI:** 10.1097/MD.0000000000034859

**Published:** 2023-08-25

**Authors:** Chen Yue, Jiahao Feng, Aili Gao

**Affiliations:** a Institute of Dermatology, Guangzhou Medical University, Guangzhou, Guangdong, China; b The Seventh Affiliated Hospital of Sun Yat-sen University, Guangzhou, Guangdong, China.

**Keywords:** drug similarity comparison, network pharmacology, psoriasis, *Shanyaotianhua* decoction, traditional Chinese medicine

## Abstract

Psoriasis is an immune-mediated inflammatory skin disease with a complex etiology involving environmental and genetic factors. Psoriasis patients often require long-term treatment. *Shanyaotianua* decoction (STT), a typical traditional Chinese medicine prescription, positively affects psoriasis, although its molecular targets remain unknown. To elucidate its molecular mechanisms, a combination of network pharmacology, bioinformatics analysis, and drug similarity comparisons were employed. Participants were separated into 3 groups: non-lesional (NL), lesions after medication (LM), and psoriasis lesion groups (LS). Based on the Gene Ontology/kyoto encyclopedia of genes and genomes enrichment analyses, the key targets were mainly enriched for biological processes (immuno-inflammatory responses, leukocyte differentiation, lipid metabolic disorders, and viral infection) with the relevant pathways (Janus kinase/signal transducers and activators of transcription and adipocytokine signaling and T-helper 17 cell differentiation), thus identifying the possible action mechanism of STT against psoriasis. Target prediction for 18 STT compounds that matched the screening criteria was performed. Then, the STT compounds were intersected with the differentially expressed genes of the psoriatic process, and 5 proteins were potential targets for STT. Based on the open-source toolkit RDKit and DrugBank database, and through molecular docking and drug similarity comparisons, spinasterol, diosgenin, and 24-Methylcholest-5-enyl-3belta-O-glucopyranoside_qt may be potential drugs for psoriasis.

## 1. Introduction

Psoriasis is an easily diagnosed common skin disorder; however, it is refractory to treatment and prone to recurrence.^[1]^ Epidemiological surveys indicate a global incidence of approximately 2% and a Chinese population incidence of 0.5%.^[2,3]^ Previous studies have implicated abnormal expression of psoriasis-susceptible genes, autoimmune disorders, obesity, and dysregulation of multiple inflammatory signaling pathways in the development of psoriasis.^[4]^ However, the underlying pathogenesis remains unclear. For patients with mild psoriasis, topical agents, including corticosteroids, vitamin D analogs, calcineurin inhibitors, and keratolytics, remain the mainstay of treatment.^[5]^ Although Western medicine has a specific curative effect, the safety of these immunotherapies poses significant concern. Traditional Chinese medicine (TCM) provides advantages including prolonged remission, stable conditions, reduced recurrence, and lower toxicity and side effects.^[6–8]^ Nonetheless, the precise mechanisms by which TCM combats psoriasis have yet to be fully elucidated, limiting its widespread adoption.

According to TCM, psoriasis is characterized by 3 predominant syndromes: blood heat, blood stasis, and blood dryness.^[6,9–12]^ Heat-clearing and blood-cooling methods are commonly employed in TCM clinical treatment for psoriasis.^[3]^ *Shanyaotianhua* decoction (STT) is a fundamental formula in Chinese medicine frequently used for psoriasis treatment. It consists of 2 representative traditional Chinese herbal medicines, namely the dry root of *Trichosanthes kirilowii Maxim (TianHuaFen*, THF) and *Dioscorea opposita (ShanYao*, SY). These herbs exert heat-clearing and toxicity-removing effects, cool the blood, and disperse blood stasis.^[6,9–12]^ Skov et al^[13]^ have highlighted that staphylococcal subclinical infections, as well as upper respiratory tract streptococcal infections, are among the causes of psoriasis. Phellodendrine, a component of THF, exhibits significant broad-spectrum antibacterial effects,^[14]^ effectively inhibiting staphylococci and enhancing the clinical treatment outcomes for patients. Psoriasis is associated with abnormalities in essential fatty acid metabolism, lymphokine release, free radical generation, and lipid peroxidation.^[15,16]^ Trillin, a secondary metabolite identified in *Dioscorea spp.*, may alleviate psoriasis symptoms in patients by increasing superoxide dismutase (SOD) activity and reducing lipid peroxidation and oxidative stress.^[17,18]^ However, the scientific basis and potential pharmacological mechanisms of STT in psoriasis treatment remain unclear and require further investigation.^[19,20]^

Network pharmacology, aligning with the “holistic” concept of TCM, utilizes a “multi-component, multi-target, multi-pathway” network construction approach. It has gained widespread application in exploring the action mechanisms of Chinese medicine pairs and compound drugs, particularly in target prediction.^[21]^ In this study, multiple algorithms and network-based computational methods were employed to predict the active components, identify various drug targets, and establish core networks of targets and elements for the treatment of psoriasis using STT. Network pharmacology and molecular docking analyses were conducted to elucidate potential mechanisms of STT and lay the groundwork for further investigation.

## 2. Materials and methods

### 2.1. Raw data collection

We obtained the corresponding clinical data and RNA-seq data of skin diseases (including 269 skin tissue samples) from the Gene Expression Omnibus dataset. The 2 cohorts used as validation datasets (GSE30999 and GSE53552) were downloaded from the Gene Expression Omnibus database (https://www.ncbi.nlm.nih.gov/geo). Non-lesion (NL) was used as a reference for differential expression analysis between the NL-psoriasis lesion (LS) groups. In total, 780 differentially expressed genes (DEGs) were identified, of which 532 were up-regulated and 248 were down-regulated. The possible occurrence of batch effects was ruled out (Supplementary Fig. 1, http://links.lww.com/MD/J578). STT compounds and target proteins were obtained from the TCM systems pharmacology database and analysis platform (TCMSP) (https://tcmspw.com/)^[22]^ and Pharmmaper server,^[23]^ respectively.

### 2.2. Differential gene expression analysis

Differential expression analysis was conducted using the R package “limma.” The screening conditions for the DEGs were |log2FoldChange| > 2.0 (NL-LS), |log2FoldChange| > 1.0 (LM-LS), and *P* value <.05. Heatmaps of DEGs were drawn using the R package “pheatmap.” Only genes with a trend consistent with the expression of clinical and treatment characteristics (|log2FC| > 1.0, *P* value < .05) in all 3 possible comparisons (NL-LS, lesions after medication (LM)-LS, and NL-LM) were considered psoriasis-specific genes.

### 2.3. GO/KEGG enrichment and GSEA analyses

Gene Ontology (GO) and kyoto encyclopedia of genes and genomes (KEGG) enrichment analyses were performed using the R packages “clusterProfiler,” “enrichplot,” and “ggplot2.” Only terms with *P*- and q-values of <.05 were considered significantly enriched. For psoriasis, GO and KEGG enrichment analyses were based on up-and down-regulated genes. For STT, “org.Hs.e.g.db” was used to convert the target protein into a gene, followed by GO and KEGG enrichment analyses. The required GMT file was downloaded from the Molecular Signatures Database (MSigDB), and gene set enrichment analysis was performed using the R package “clusterProfiler” (|log2FC| > 0.5, p.adj < 0.05).

### 2.4. Active compound screening

All the chemical ingredients of STT were obtained from TCMSP. STT is composed of 2 TCM herbs, THF and SY. The active STT compounds were mainly filtered with oral bioavailability, drug-likeness, and Caco-2 permeability, whereas STT compounds with oral bioavailability ≥20%, drug-likeness ≥0.18, and Caco-2 permeability ≥−0.04 were preserved.^[24]^ The STT compounds that met the screening criteria are shown in Supplementary Table 1, http://links.lww.com/MD/J579.

### 2.5. Network pharmacology analysis

Cytoscope 3.7.0 software was employed to construct the active compound-target network, and compounds with a larger number of nodes were selected for key analysis. The STT compound 3D structure was acquired from TCMSP. The PharmMapper service (http://lilab.ecust.edu.cn/pharmmapper/) utilized the compounds’ pharmacophore as the probe molecule recognition group to predict target proteins. A stringent threshold (z-score = 1.0) was selected, and all protein targets above this threshold were extracted for further analysis.^[25]^ Existing drug therapeutic targets were retrieved from the DrugBank database (https://go.drugbank.com/).

### 2.6. Drug similarity analysis

The “full database” document was downloaded from the DrugBank database,^[26]^ which contains all the drug details. During document processing, the drug information with a “small molecule” type was selected, and the protein category drug was removed as the small molecule compounds in STT were prioritized. The top 10 similar drugs were identified by comparing active STT compounds to small-molecule drugs in the DrugBank database. On the basis of the 2D and 3D structures of the compounds, the RDkit tool (Python environment, https://www.python.org) was used to build compound descriptors and PubChem fingerprints (https://pubchem.ncbi.nlm.nih.gov) and to calculate the structural similarities between compounds.^[27]^

### 2.7. Molecular docking

According to the results from Network analysis of active STT compounds, the 3D structure of proteins: serum S100 calcium-binding protein A9(S1000A9) (PDB ID:4GGF, Uniprot: P28783), GM2 activator protein (GM2A) (PDB ID:2AG4, Uniprot: Q60648), remote electrical neuromodulation (REN) (PDB ID:3K1W, Uniprot: P00797), macrophage elastase (MMP12) (PDB ID:1Y93, Uniprot: P399900), and Retinol binding protein 4 (RBP4) (PDB ID:5NU2, Uniprot: P02753) were selected and downloaded from the Protein Data Bank (PDB) database (https://www.rcsb.org/). RMSD and RMSF values were calculated using the Python smartools VMD.^[28]^ The structure of the core gene protein was dehydrated and hydrogenated, and pymol 2.3 was used to insert hydrogen atoms. The targets were set to rigid and saved in a PDBQT file format using AutoDock Tools 1.5.6. The Vina software was applied to do molecular docking. Based on the top minimal binding energy of each target, STT compounds were chosen for further analysis of their binding mode, binding affinity, and critical interactions using pymol2.3.^[25]^

## 3. Results

### 3.1. Identification of psoriasis progression DEGs by transcriptome analysis

**Figure 1.**
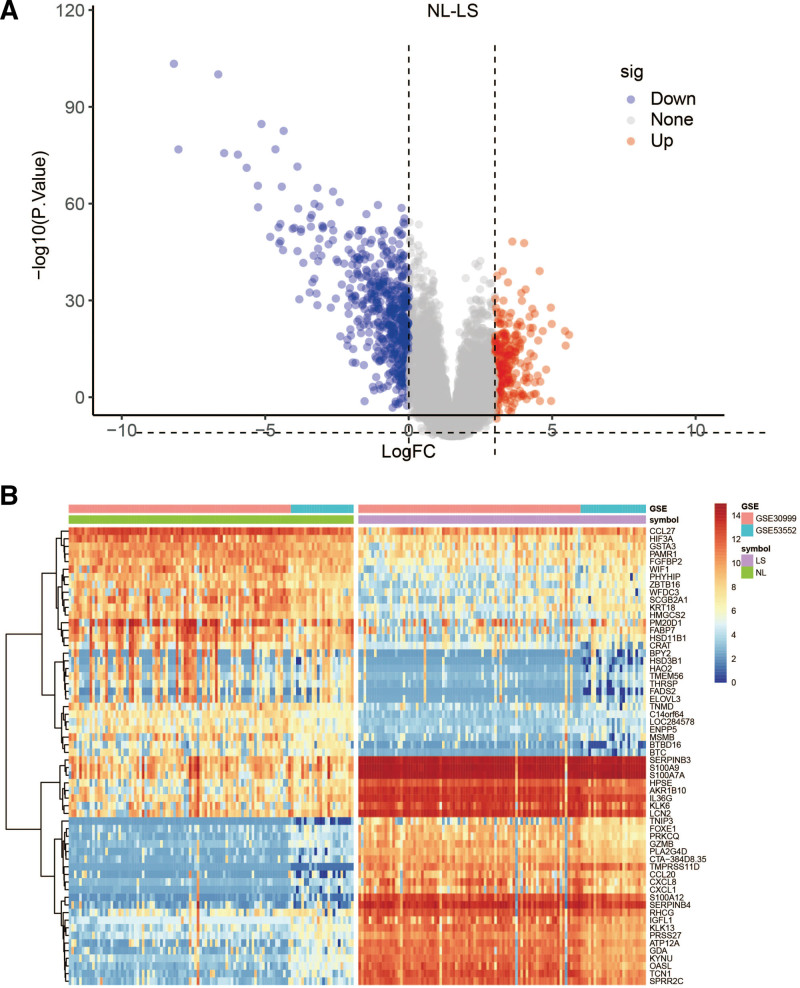
Volcano plots and heatmaps of DEGs in NL-LS. (A) Overall distribution and number of up-and down-regulated genes. (B) Heatmap for DEGs generated by comparison in NL and LS. DEGs were detected by Wilcoxon rank sum test (q < 0.05 and log2FC > 0.3). The row represents the gene, while the column identifies the samples that are not shown in the plot. DEGs = differentially expressed genes, LS = psoriasis lesion, NL = non-lesion.

NL was used as a reference for differential expression analysis between the NL-LS groups. In total, 780 DEGs were identified, of which 532 were up-regulated and 248 were down-regulated (Supplementary Table 2, http://links.lww.com/MD/J580). Figure 1 shows DEG expression in NL-LS, and Supplementary Figure 2, http://links.lww.com/MD/J581 shows DEG expression in NL-LM-LS.

### 3.2. GO/KEGG enrichment analysis for NL-LS

#### 3.2.1. GO enrichment analysis of NL-LS.

The degrees of NL-LS progression were categorized according to the up-and down-regulated expression, and GO enrichment analysis was performed (*P* < .01). In total, 137 GO entries of up-regulated genes, including 134 biological processes (BPs) and 3 cellular components, and 560 GO entries of down-regulated genes, including 462 BPs, 79 molecular functions (MFs), and 19 cellular components, were obtained.

**Figure 2.**
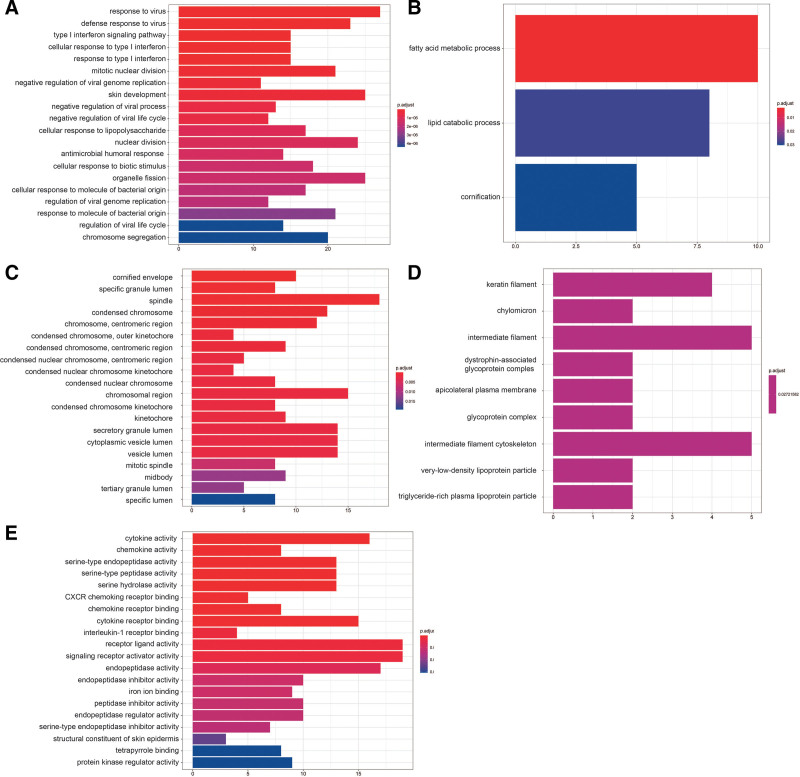
GO enrichment analysis of down-/up-regulated DEGs. DEGs = differentially expressed genes, GO = gene ontology.

The BP entry (p.adj < 0.01) of down-regulated genes in NL-LS mainly included type I interferon (IFN) signaling pathway, response to virus, skin development, and cellular response to type I IFN (Fig. 2A), suggesting that type 1 interferons (IFNα and IFNβ), key cytokines that activate autoimmunity during viral infection, may play an indispensable role in initiating psoriasis during skin injury.^[29]^ The enriched GO-BP terms of up-regulated genes (p.adj < 0.01) are primarily associated with fatty acid metabolic processes, lipid catabolic processes, and cornification (Fig. 2B). This result corresponds to the pathological phenotype of LS. Obesity is strongly associated with psoriasis onset and exacerbation and is defined as a white adipose tissue expansion. Various mediators secreted by the adipose tissue induce a low-grade inflammatory state, contributing to psoriasis pathogenesis.^[16]^ Figure 2C and D show GO-CC enrichment analysis results (p.adj < 0.01). GO-MF enrichment results indicated that the down-regulated genes (*P* < .01) were mainly mapped to receptor ligand, signaling receptor activator, cytokine activities, cytokine receptor binding, and other immune stress-related pathways, similar to the BP results (Fig. 2E).

#### 3.2.2. KEGG enrichment analysis.

**Figure 3.**
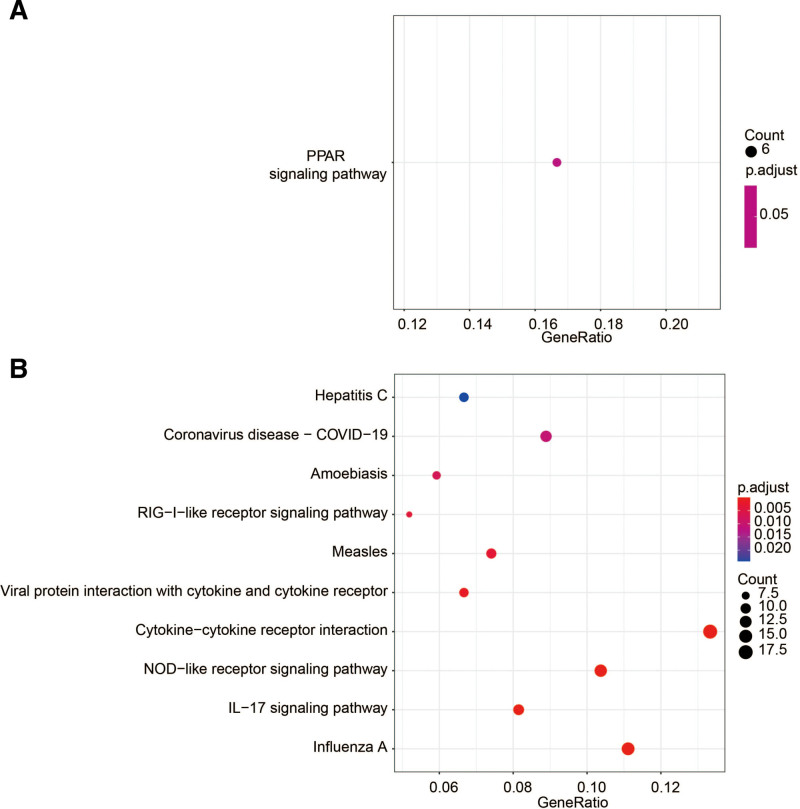
KEGG analysis of the up-/down-regulated DEGs involved in LS. DEGs = differentially expressed genes, KEGG = Kyoto encyclopedia of genes and genomes.

Up-regulated gene expression enrichment (p.adj < 0.05) was observed in the peroxisome proliferator-activated receptor (PPAR) signaling pathway in the NL-LS group (Fig. 3A). PPARs are ligand-activated transcription factors that regulate lipid metabolism and energy homeostasis. Accumulating evidence suggests that PPARs play a role in genomic pathways, including regulating cell growth, apoptosis, and differentiation. Given that psoriasis is an inflammatory skin disorder characterized by epidermal hyperproliferation and abnormal keratinocyte differentiation, PPARs may be potential targets for treatment.^[30]^ Down-regulated genes were enriched in IL-17 signaling pathway, cytokine-cytokine receptor interaction, influenza A, and nucleotide oligomerization domain like receptor signaling pathways (Fig. 3B). An extensive amount of evidence now places IL-17 signaling pathway as a key player in psoriasis pathogenesis.^[31]^ IL-17 is mainly released by human T-helper 17 (Th17) cells and an important role of IL17 appears to be the regulation of local inflammation through the upregulation of other pro-inflammatory cytokines and chemokines. Recently, the FDA approved multiple highly effective psoriasis therapies that disrupt interleukin-17 (secukinumab, ixekizumab, and brodalumab) and interleukin-23 (guselkumab and tildrakizumab) signaling in the skin, all with promising therapeutic results.^[32]^ Cytokine-cytokine receptor interaction and nucleotide oligomerization domain like receptor signaling pathways regulate proliferation and differentiation.This is consistent with the clinical features described in other published studies.^[33]^ KEGG enrichment analysis results were consistent with that of GO enrichment, enhancing the reliability of GO enrichment results and providing more evidence that inflammation, hyperproliferation and lipid metabolism disorders as the cause of LS.

### 3.3. Target of compounds prediction

**Figure 4.**
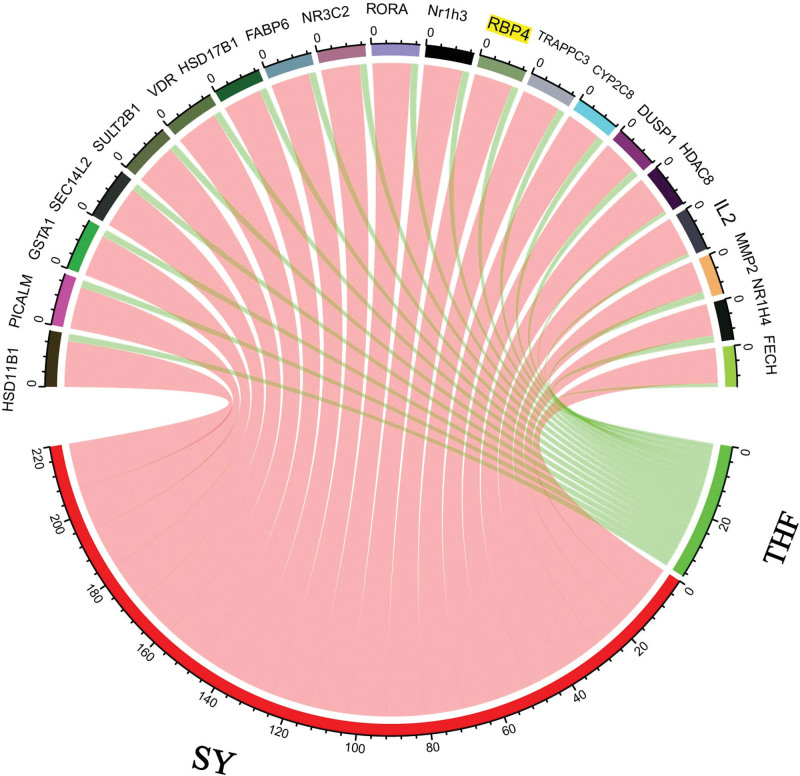
The chord diagram of STT and main target proteins. STT = *Shanyaotianua* decoction.

The interaction between the predicted targets and drug-like compounds (Supplementary Table 1, http://links.lww.com/MD/J579) and the statistical analysis of the predicted targets are shown in Figure 4. The number of correspondence between compounds and predicted targets was proportional relationship with its action in psoriasis and these were used to create chord diagrams that show the strength of these relationships (in the size of the chords) and the herbs to which they belong (green is THF, red is SY). The target proteins frequently targeted by compounds may be the specific STT targets for treating psoriases, such as RBP4, PICALM, GSTA1, CYP2C8, DISP1, HDAC8, IL-2, MMP2, NR1H4, SEC14L2, SULT2B1, HSD11B1, VDR, HSD17B1, FABP6, NR3C2, RORA, Nr1h3, TRAPPC3, and FECH. Based on our results in Figure 4, we strongly favor that RBP4 likely represents the most important potential target for treating psoriasis.

### 3.4. GO/KEGG enrichment and GSEA analyses for STT targets

**Figure 5.**
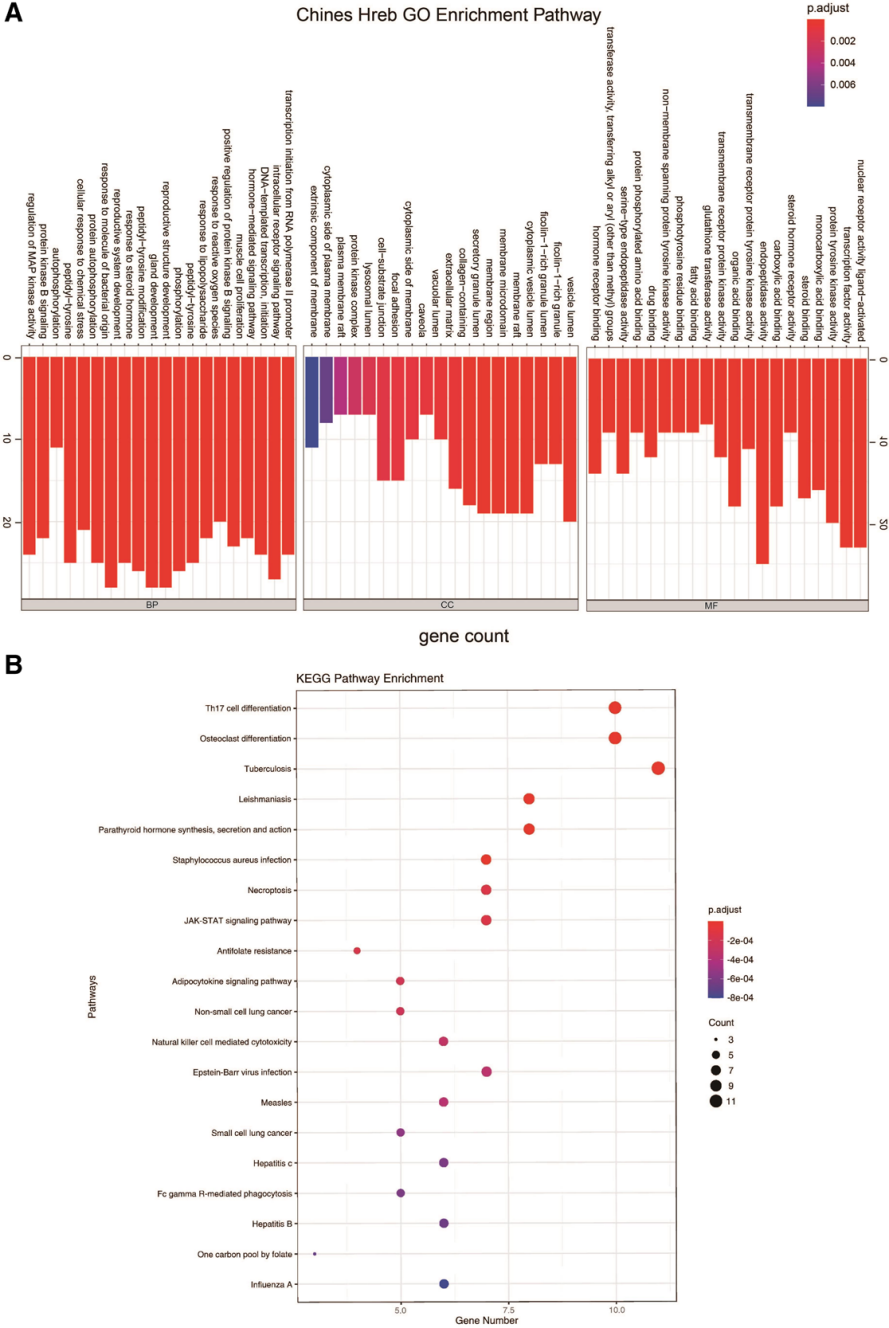
GO and KEGG analysis of the STT target proteins. GO = gene ontology, KEGG = Kyoto encyclopedia of genes and genomes, STT = *Shanyaotianua* decoction.

GO analysis was performed on the STT target gene and took the *P* < .05 of GO annotation enrichment as the significance threshold. Figure 5A shows the top 20 significantly enriched GO-BP terms. GO-BP enrichment analysis indicated that numerous targets are included in various BPs engaged in the intracellular receptor signaling pathway, reproductive structure development, gland development, reproductive system development, and cellular response to chemical stress. Analysis of GO-CC enrichment showed that STT mainly acted on the vesicle lumen, cytoplasmic vesicle lumen, and membrane raft, among others. GO-MF enrichment analysis demonstrated that STT targets were involved in protein tyrosine kinase activity, ligand-activated transcription factor activity, nuclear receptor activity, and endopeptidase activity, among others.

In addition, the KEGG pathway enrichment analysis of the STT targets (*P* < .05) revealed significant enrichment in inflammation-related pathways such as the Janus kinase/signal transducers and activators of transcription (JAK-STAT) and adipocytokine signaling pathways, Th17 cell and osteoclast differentiation, leishmaniasis, parathyroid hormone synthesis, secretion and action, staphylococcus aureus infection, and necroptosis, among others (Fig. 5B).

**Figure 6.**
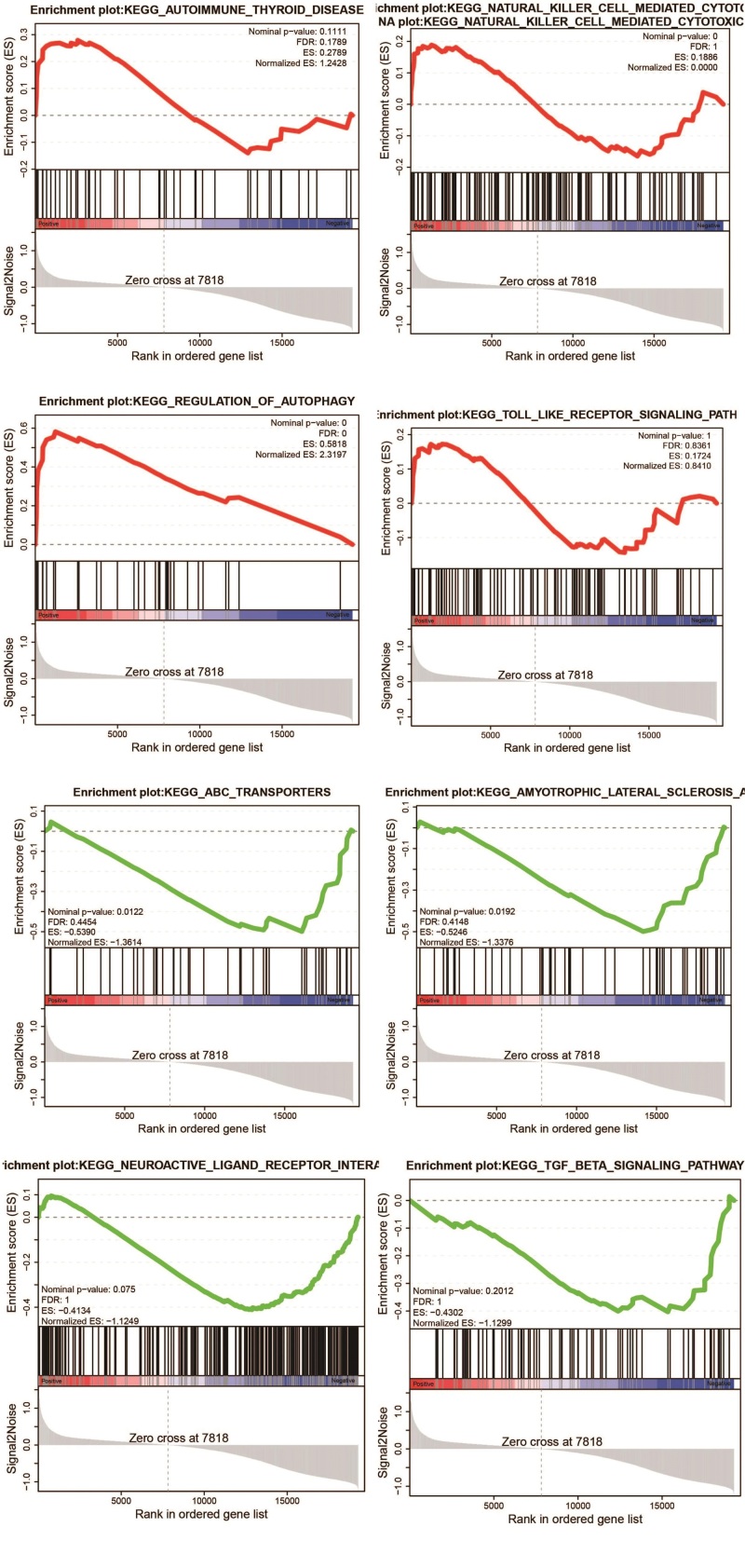
GSEA analysis of the STT target proteins. GSEA = gene set enrichment analysis, STT = *Shanyaotianua* decoction.

To comprehensively understand the relationship between up- and down-regulated genes and psoriasis biology, gene set enrichment analysis was used for marker functional enrichment based on the DEGs between NL and LS (Fig. 6). The up-regulated genes were enriched in natural killer cell regulation, autophagy, Toll-like receptor signaling, and autoimmunity. Down-regulated genes were enriched in ATP-binding cassette (ABC) transporters, TGF-β and neuroreceptors. TGF-β pathway, enriched in down-regulated genes, has been identified as an important recovery index in psoriasis.^[34]^ However, inflammation-related pathways, which are common in psoriasis and hinder recovery from psoriasis, are up-regulated and activated.^[35]^

### 3.5. Network analysis of active STT compounds

**Figure 7.**
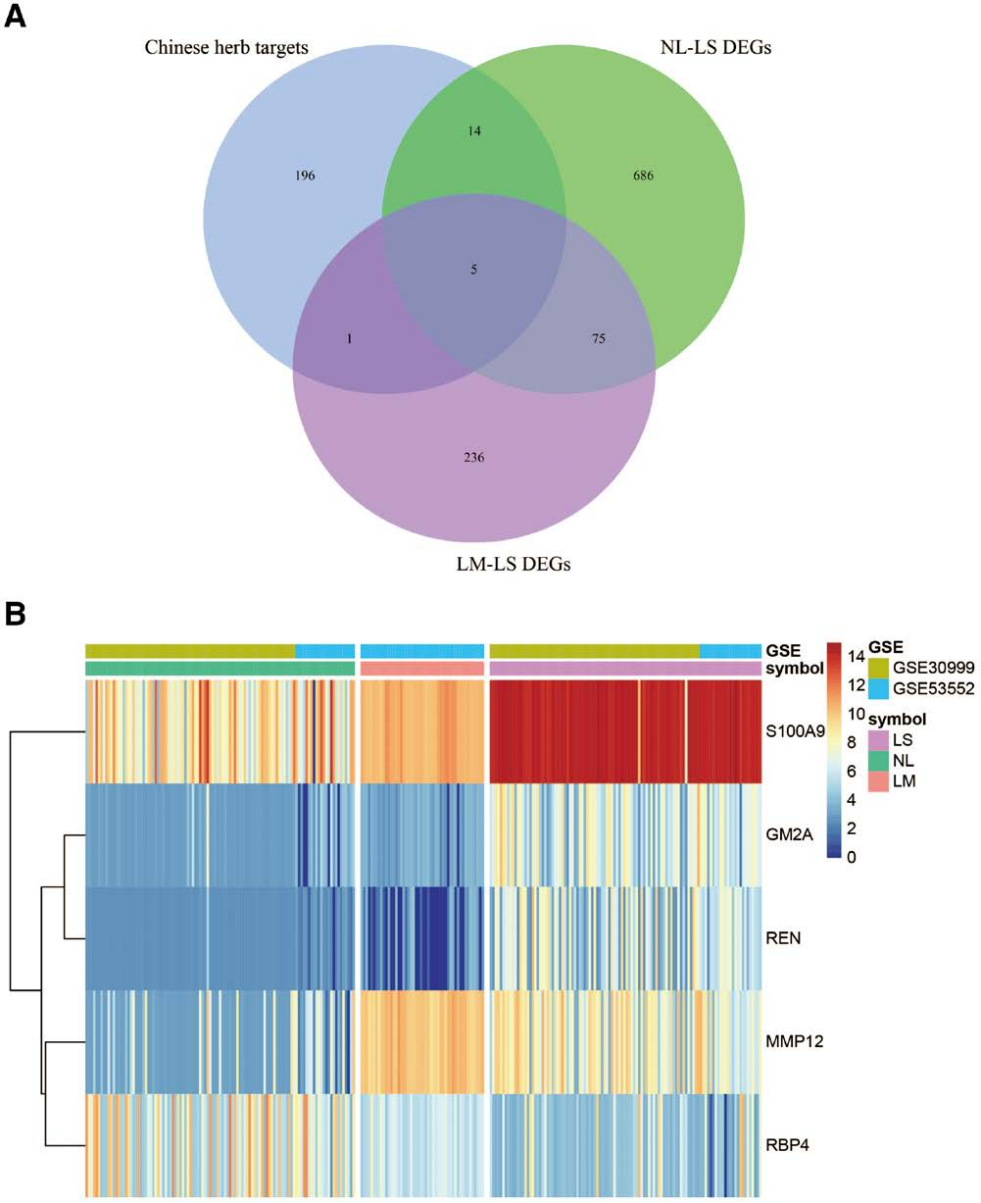
Intersection of NL-LM-LS and STT target proteins Venn Diagram and heatmap. STT = Shanyaotianua decoction, LM = lesions after medication, LS = psoriasis lesion, NL = non-lesion.

Five target proteins were identified by intersecting the predicted STT target with the intensity of all psoriatic stages (Fig. 7A and B). STT targets in NL-LM-LS are S1000A9, GM2A, RBP4, MMP12, and REN. Several studies support the important role of S100A9 in psoriasis. S100A9 was found in the lesional skin of psoriatic patients; however, its expression was low in the NL skin sample from the same individual.^[36]^ Consistently, S100A8/A9 serum levels are elevated in psoriatic patients, and elevated S100A8/A9 serum levels track disease activity, suggesting that these S100 proteins are potential mediators in psoriasis.^[37]^ GM2A is a lipid transfer protein that stimulates the enzymatic processing of gangliosides and activates T cells via lipid presentation while acting as a gene encoding lysosomal enzyme.^[38]^ Katarzyna et al^[39,40]^ demonstrated that GM2A was up-regulated in an in vitro psoriatic model established using Hacat cells. MMP12 (human macrophage metalloproteinase) has been identified as an elastolytic metalloproteinase secreted by inflammatory macrophages^[41]^; the levels of MMP12 messenger RNA (mRNA) were 17-fold higher in psoriatic lesions than in NL skin.^[42]^ RBP4, a protein in the lipocalin family, has been associated with various metabolic complications, such as metabolic syndrome, obesity, and insulin resistance, and is closely associated with psoriasis.^[43]^ Previous clinical studies have identified increased serum levels of RBP4 in patients with psoriasis compared to controls,^[44]^ suggesting that RBP4 may have a protective effect against chronic inflammation and psoriatic complications. Therefore, these may be the potential targets by which STT combats psoriasis.

**Figure 8.**
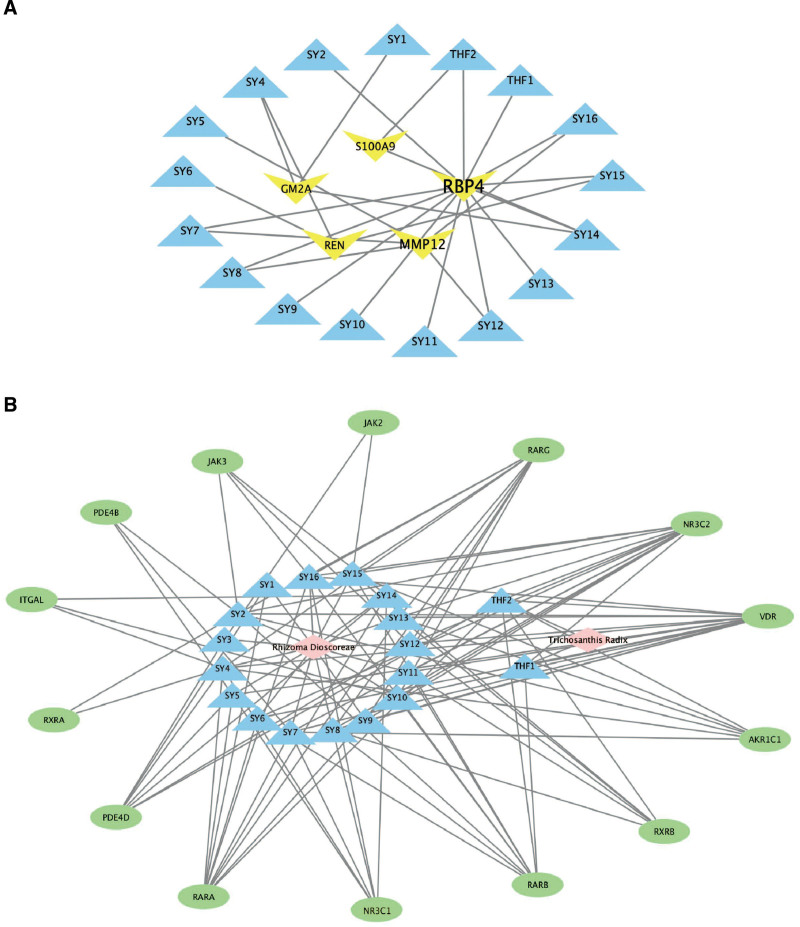
The overall interactive network diagram (herbs, active compounds, target proteins). (A) Target proteins are linked to their corresponding diseases and those diseases are linked to the corresponding disease categories they belong to. The node size and the number of the line have a direct proportional (positive) relationship with the therapeutic effect. The blue triangles represent the compounds of herbs; the yellow darts represent clinical drug targets. (B) The pink diamonds represent herbs; the blue triangles represent the compounds of herbs; the ovals represent the intersecting target proteins in treating psoriasis.

Small-molecule compounds (Fig. 8A), such as SY14 and THF2, with numerous targets and a therapeutic effect on the targets, were included in the follow-up analysis to interpret the mechanism by which they combat psoriasis. The compounds with many targets and the target proteins of clinical drugs were analyzed on the basis of the results of our previous findings (Fig. 8A). SY4, SY7, SY8, SY12, SY14, SY15, SY16, and THF2, among others, met the requirements of the 2 parts of the protein-protein interaction analysis. After the intersection process, we found several overlapping targets between small-molecule compounds and key targets in psoriasis. They are retinoic acid receptor alpha (RARA), retinoic acid receptor beta (RARB), retinoid X receptor beta (RXRB), NR3C2, vitamin D3 receptor (VDR), aldo-keto reductase (AKR1C1) and phosphodiesterase-4D(PDE4D), which meant that these targets might be the key targets for STT treating psoriasis (Fig. 8B).

### 3.6. Similarity analysis of predicted active compounds

**Figure 9.**
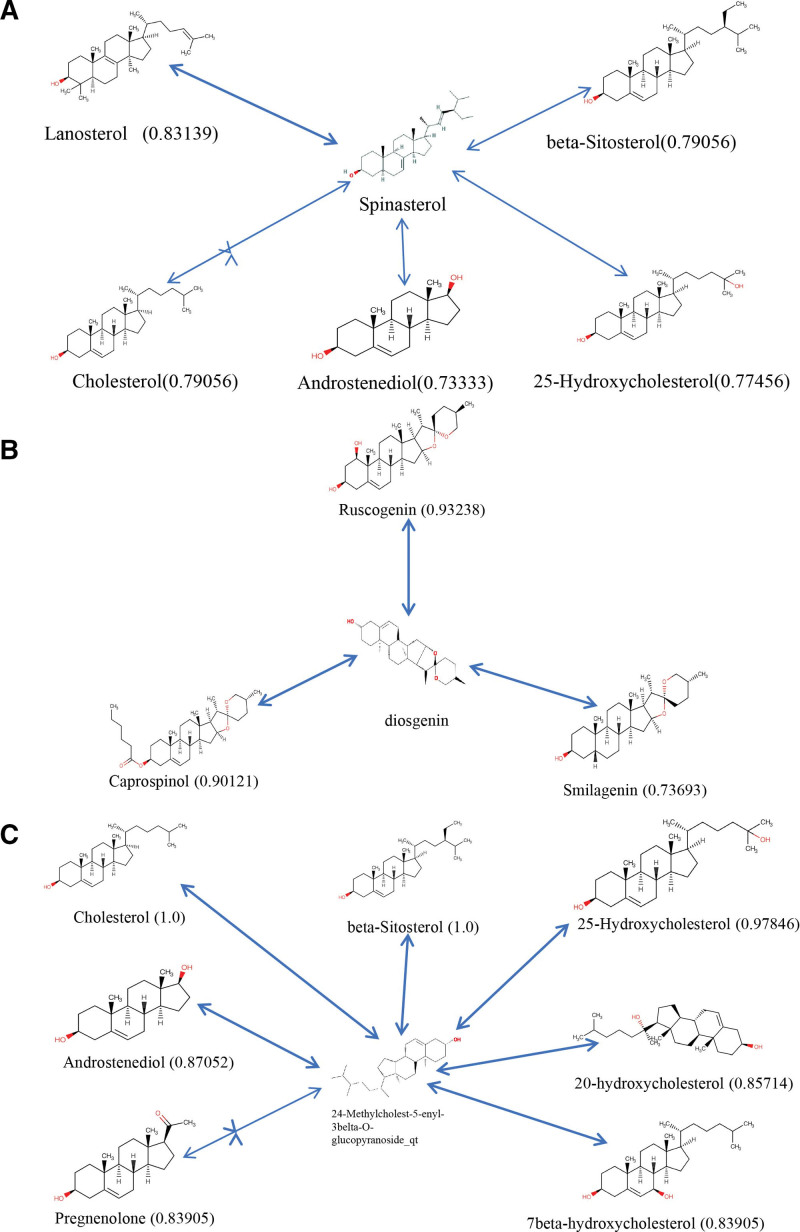
Comparability of compounds and drugs from the DrugBank.

The RDkit tool ranked the small molecular compounds in the DrugBank database and recommended the top 10 drug compounds with similarities. The drug similarities included spinasterol and lanosterol (0.83139), cholesterol (0.79056), beta-sitosterol (0.79056), 25-Hydroxycholesterol (0.77456), and androstenediol (0.73333) (Fig. 9A). Figure 9B illustrates the similarities between diosgenin and ruscogenin (0.93238), caprospinol (0.90121), and smilagenin (0.73693). Figure 9C demonstrates that 24-Methylcholest-5-enyl-3belta-O-glucopyranoside_qt(24-M5) exhibited high similarity to cholesterol (1.0), beta-sitosterol (1.0), 25-Hydroxycholesterol (0.97846), AED (0.87052), 20-HC (0.85714), pregnenolone (0.83905), and 7beta-HC (0.83905). These compounds may serve as the foundation for the therapeutic effect of STT on psoriasis.

### 3.7. Molecular docking

**Figure 10.**
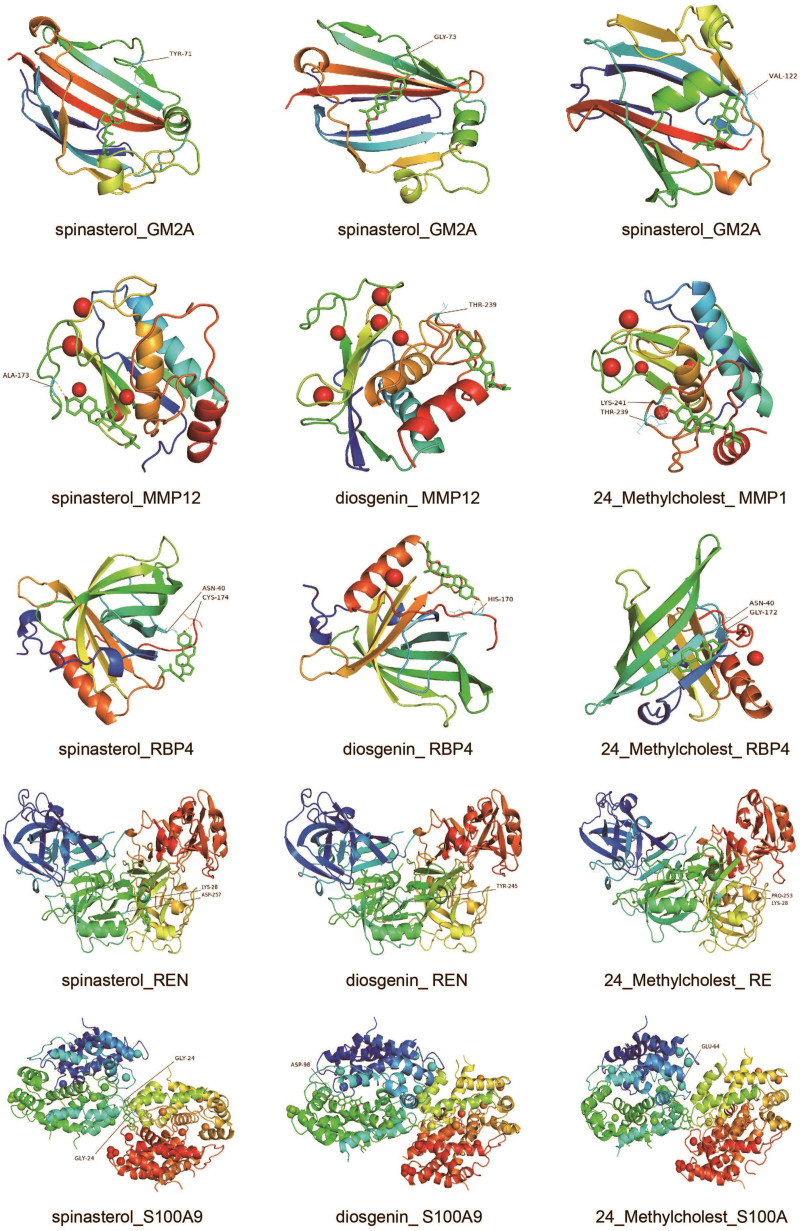
Molecular docking models of putative interactions with target proteins.

In Supplementary Table 3, http://links.lww.com/MD/J582, the docking results of the active compounds with *GM2A, MMP12*, RBP4, REN, and S100A9 are presented. As shown in the analysis of binding energy results, the target proteins formed stable conformations with STT active compounds. The core targets, such as GM2A, MMP12, RBP4, REN and S100A9, were chosen for molecular docking with STT active compounds (Fig. 10). Compound spinasterol exhibited the lowest binding energy with GM2A (−9.3 kcal/mol), MMP12 (−8.09 kcal/mol), RBP4 (−8.03 kcal/mol), REN (−8.43 kcal/mol), and S100A9 (−7.29 kcal/mol). Diosgenin showed the lowest binding energy with GM2A (−8.99 kcal/mol), MMP12 (−9.2 kcal/mol), RBP4 (−7.97 kcal/mol), REN (−8.73 kcal/mol), and S100A9 (−7.77 kcal/mol). 24_Methylcholest had the lowest binding energy with GM2A (−6.95 kcal/mol), MMP12 (−8.24 kcal/mol), RBP4 (−7.4 kcal/mol), REN (−7.02 kcal/mol), and S100A9 (−7.41 kcal/mol). Binding energy analysis showed that STT active compounds formed stable conformations with the target proteins. The docking analysis between the selected compounds and target proteins is shown in Figure 10. Compound spinasterol bound to GM2A, forming hydrogen bond interactions with residues TYR-71; MMP12, forming hydrogen bond interactions with residues ALA-173; and RBP4, forming hydrogen bond interactions with residues ASN-40 and CYS-174. When binding to GM2A, diosgenin formed hydrogen bonds with residues GLY-73. Furthermore, when binding to RBP4, diosgenin formed hydrogen bond interactions with residues HIS-170. In addition, compound 24_Methylcholest bound to S100A9, forming hydrogen bond interactions with residue GLU-64; to REN, forming hydrogen bond interactions with residues PRO-253 and LYS-28; and to RBP4, forming hydrogen bond interactions with residue ASN-40 and GLY-172.

## 4. Discussion

Psoriasis is a chronic and complex dermatological disorder encompassing various subtypes, characterized by epidermal hyperplasia, excessive proliferation of keratinocytes, activation of the immune system, and genetic susceptibility. This condition imposes a significant burden on patients’ daily lives.^[33,45–47]^ Moreover, conventional treatment methods have proven inadequate in achieving the desired therapeutic effects for many individuals with psoriasis. In recent decades, TCM has emerged as a promising approach for treating psoriasis, owing to its numerous advantages. The 2015 edition of the Chinese Pharmacopoeia recommends specific Chinese herbal compound formulas tailored to the 3 main patterns of psoriasis: blood heat pattern, blood dryness pattern, and blood stasis pattern.^[48]^ The efficacy of these formulations has been extensively investigated through clinical and experimental studies.^[49,50]^ Furthermore, controlled trials have demonstrated that combining TCM with conventional therapy yields superior therapeutic outcomes in the treatment of psoriasis.^[51]^ While SY and THF are commonly used conventional treatments for progressive psoriasis, STT has shown significant clinical efficacy in psoriasis treatment.^[8,9,52,53]^ However, the core targets and active ingredients of STT remain largely unknown, posing a challenge to its further development and clinical application. Therefore, this study employs network pharmacology and molecular docking techniques to elucidate the mechanisms underlying the therapeutic effects of STT in psoriasis.

By gene expression profiles and bioinformatics analysis, the disease patterns and key targets of psoriasis were identified. The most important targets were S100A9, IL-36γ, and CXCL1. Loss of S100A8-S100A9 prevents psoriasis-like symptoms, suggesting that this up-regulation may trigger disease initiation.^[54]^ IL-36γ played a specific role in psoriasis. Its protein expression level was approximately 3 times higher in psoriatic lesions than in other inflammatory skin disorders.^[55]^ IL-36γ expression correlates with disease activity and decreases during TNF-α treatment, improving disease manifestation.^[56]^ CXCL1 is a member of the CXC subfamily of chemokines, which is up-regulated in the lesional skin of psoriatic patients and down-regulated to normal levels by biologics targeting TNF-α or IL-17A.^[57,58]^ The BPs involved were the response to IFN and granulocyte chemotaxis. According to the pathway analysis and GO terms in this study, DEGs were enriched in the “defense response to virus,” “mitotic nuclear division,” “response to type I IFN,” “fatty acid metabolic process,” and “skin development,” consistent with extensive previous studies on psoriasis. These results suggest that psoriasis pathogenesis may be closely related to viral infection and lipid metabolism.^[15,59,60]^

The network pharmacology results revealed that the key targets of active STT components, namely GM2A, REN, MMP12, RBP4, and S100A9, are primarily associated with psoriasis, potentially serving as the basis for the therapeutic effectiveness of STT. The GO-BP and KEGG enrichment analysis of the key targets revealed that STT exerts its therapeutic effect on psoriasis through multiple BPs and signaling pathways, including Th17 cell differentiation, JAK-STAT, mitogen-activated protein kinase, and adipocytokine signaling pathways. This study suggests that the immune-enhancing and anti-inflammatory properties of STT may be attributed to their influence on cytokine activity and the processes of cytokine receptor binding. Based on the analysis of drug similarity, the key bioactive components in STT are spinasterol, diosgenin, and 24-M5. Spinasterol, a phytosterol, has been reported to exhibit anti-inflammatory properties by inhibiting the production of pro-inflammatory cytokines and modulating immune responses.^[61]^ Diosgenin, a steroidal saponin, has demonstrated anti-inflammatory and immunomodulatory effects, potentially influencing immune cell functions and inflammatory pathways.^[62]^ 24-M5, a synthetic derivative of cholesterol, has shown anti-inflammatory properties and the ability to modulate immune responses.^[63]^ These compounds have the potential to modulate key pathways involved in the pathogenesis of psoriasis, including the regulation of pro-inflammatory cytokines, activation of immune cells, and abnormal keratinocyte proliferation. The identification of these structural similarities highlights the potential of the STT compounds as new therapeutic candidates for psoriasis. Further research is necessary to investigate their specific mechanisms of action, evaluate their efficacy in preclinical and clinical settings, and assess their safety profiles. Clinical trials involving these compounds could offer valuable insights into their therapeutic potential and facilitate the development of targeted and personalized treatment approaches for patients with psoriasis.

However, certain limitations are noteworthy. Firstly, our research relies on public databases that may contain incomplete data parameters, resulting in potential errors and deviations. Secondly, the active ingredients of TCM exhibit activity on multiple targets and pathways, posing challenges in elucidating their mechanisms of action. Moreover, while our study presents a plausible direction for future in vitro and in vivo investigations, it lacks experimental validation. Therefore, larger clinical trials and animal studies are necessary to substantiate the efficacy of STT.

## 5. Conclusion

In conclusion, this study is the first to perform gene enrichment analysis, target prediction, network analysis, integrate active components, and utilize a network pharmacology and molecular docking techniques approach (flowcharts are shown in Supplementary Fig. 3, http://links.lww.com/MD/J583) to systematically elucidate the molecular and pharmacological mechanisms underlying the amelioration of psoriasis by STT. The network pharmacology results revealed that the primary targets of the active ingredients in STT were S100A9, GM2A, REN, MMP12, and RBP4, all of which were primarily associated with psoriasis. The potential therapeutically active compounds in STT include spinasterol, diosgenin, and 24-M5. These findings may provide the basis for the therapeutic effectiveness of STT. However, further experiments and additional clinical trials are necessary to validate the above findings.

## Author contributions

**Formal analysis:** Chen Yue.

**Project administration:** Chen Yue.

**Software:** Jiahao Feng.

**Supervision:** Aili Gao.

**Writing – original draft:** Chen Yue.

**Writing – review & editing:** Aili Gao.

## Supplementary Material












