# Supplementary figures and images for: A network pharmacology and molecular docking investigation on the mechanisms of Shanyaotianhua decoction (STT) as a therapy for psoriasis
Source: Medicine (Baltimore). 2023 Aug 25;102(34):e34859. doi: 10.1097/MD.0000000000034859 (PMC10470816; doi:10.1097/MD.0000000000034859)

**Supplementary Figure 1** | Removal of batch effects in different datasets and PCA analysis

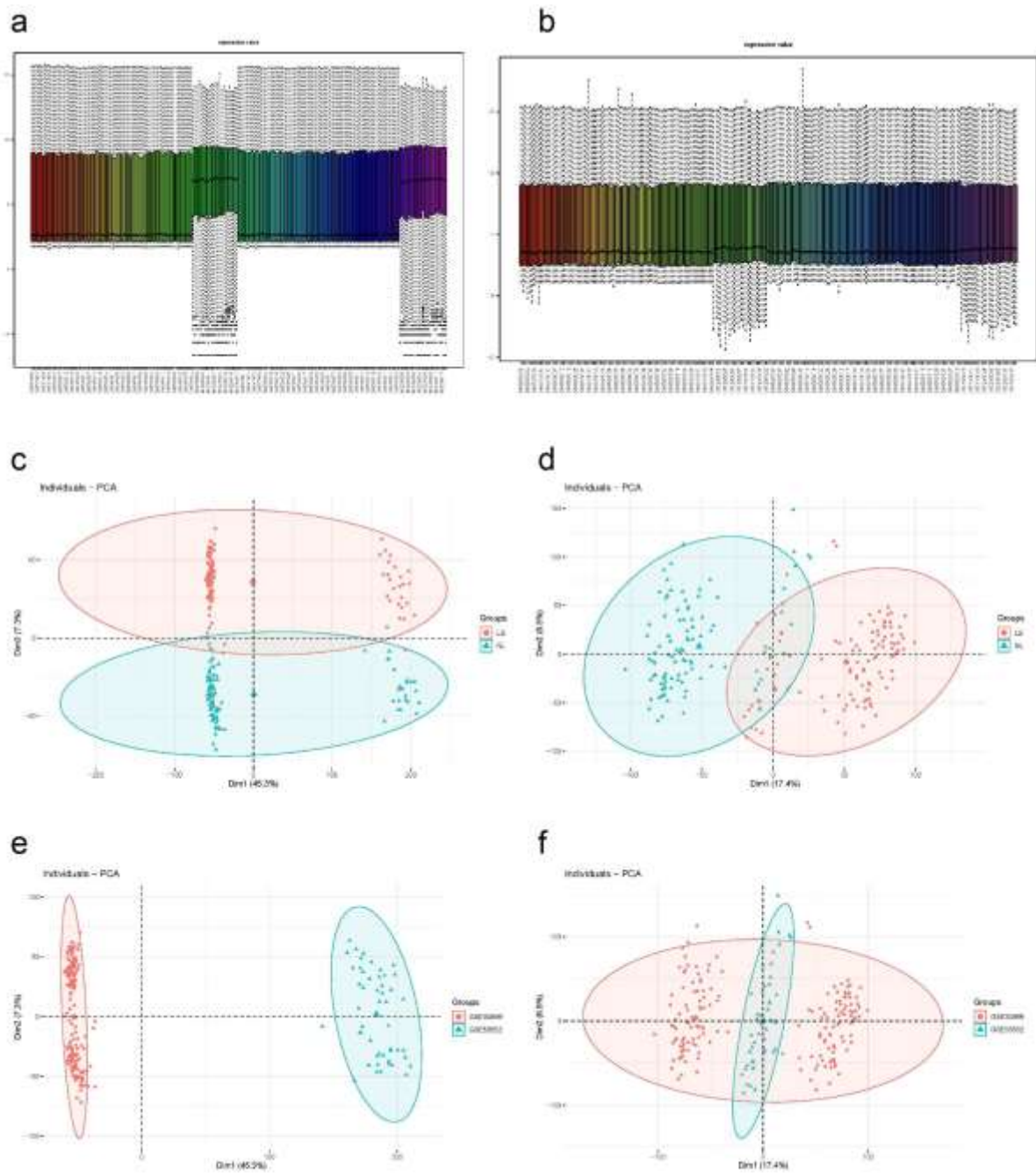

Supplement: Supplementary file 1 [file medi-102-e34859-s001.pdf]

**Supplementary Figure 2** | The expression level of common up- and down-regulated DEGs in NL, LM and LS.

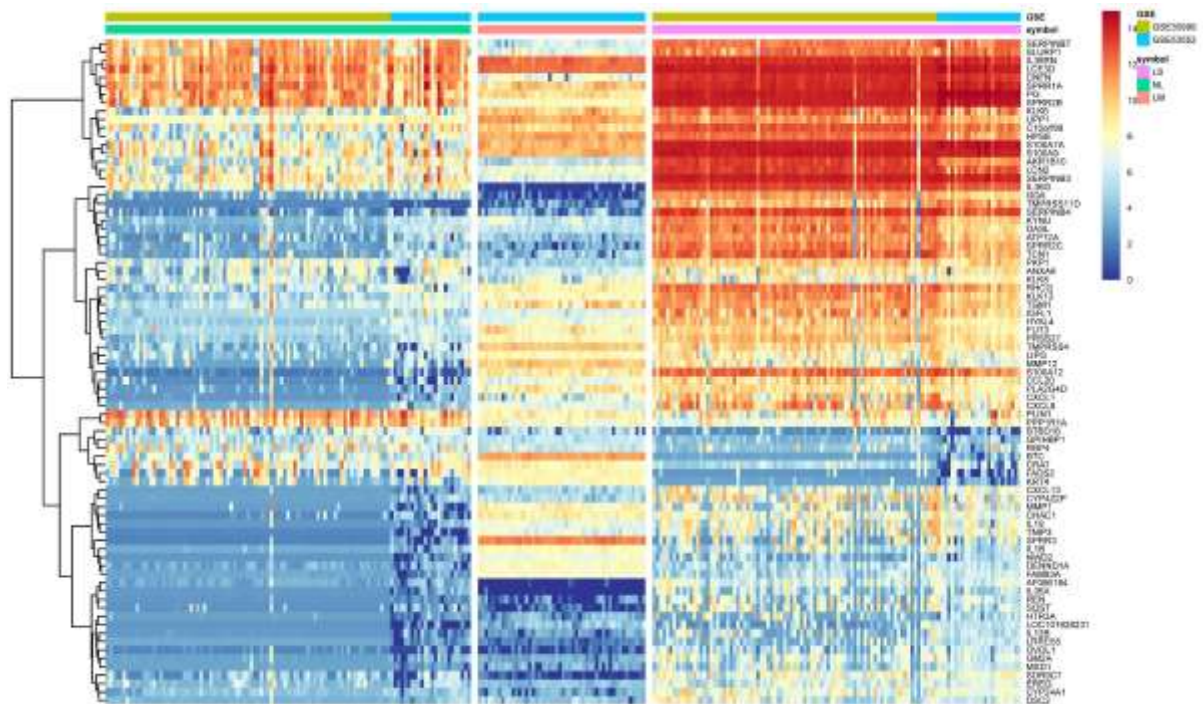

Supplement: Supplementary file 4 [file medi-102-e34859-s004.pdf]
