# Supplementary material for: A network pharmacology and molecular docking investigation on the mechanisms of Shanyaotianhua decoction (STT) as a therapy for psoriasis
Source: Medicine (Baltimore). 2023 Aug 25;102(34):e34859. doi: 10.1097/MD.0000000000034859 (PMC10470816; doi:10.1097/MD.0000000000034859)
Supplement: Supplementary file 2 [file medi-102-e34859-s002.pdf]

number	compound	structure	OB（%）	Caco-2	DL	herb
THF1	Schottenol	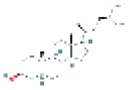	37.42	1.33	0.75	Trichosanthis Radix
THF2	Spinasterol	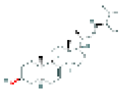	42.98	1.44	0.76	Trichosanthis Radix
SY1	piperlonguminine	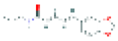	30.71	0.95	0.18	Rhizoma Dioscoreae
SY2	Methylcimicifugoside_qt	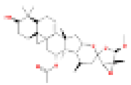	31.69	0.21	0.24	Rhizoma Dioscoreae
SY3	(-)-taxifolin	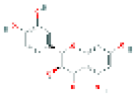	60.51	-0.24	0.27	Rhizoma Dioscoreae
SY4	hancinol	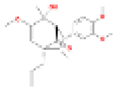	64.01	0.53	0.37	Rhizoma Dioscoreae
SY5	Kadsurenone	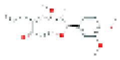	54.72	0.82	0.38	Rhizoma Dioscoreae
SY6	Denudation	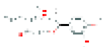	61.47	0.9	0.38	Rhizoma Dioscoreae
SY7	hancinone C	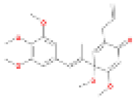	59.05	0.74	0.39	Rhizoma Dioscoreae
SY8	Doradexanthin	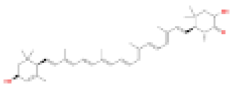	38.16	0.52	0.54	Rhizoma Dioscoreae

SY9	CLR	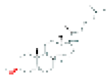	CAR87	1.43	0.68	Rhizoma Dioscoreae
SY10	campesterol	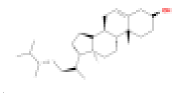	37.58	1.34	0.71	Rhizoma Dioscoreae
SY11	24-Methylcholest-5-enyl-3belta-O-glucopyranoside_qt	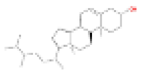	37.58	1.33	0.72	Rhizoma Dioscoreae
SY12	Stigmasterol	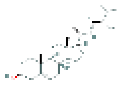	43.83	1.44	0.76	Rhizoma Dioscoreae
SY13	Isofucosterol	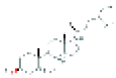	43.78	1.36	0.76	Rhizoma Dioscoreae
SY14	AIDS180907	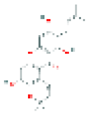	45.33	0.73	0.77	Rhizoma Dioscoreae
SY15	diosgenin	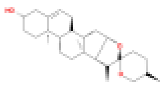	80.88	0.82	0.81	Rhizoma Dioscoreae
SY16	Dioscoreside C_qt	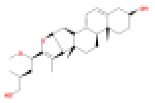	36.38	0.39	0.87	Rhizoma Dioscoreae

Supplementary Table 1 Properties of Effective Drug-like Compounds of STT
