# Supplementary material for: A network pharmacology and molecular docking investigation on the mechanisms of Shanyaotianhua decoction (STT) as a therapy for psoriasis
Source: Medicine (Baltimore). 2023 Aug 25;102(34):e34859. doi: 10.1097/MD.0000000000034859 (PMC10470816; doi:10.1097/MD.0000000000034859)
Supplement: Supplementary file 3 [file medi-102-e34859-s003.pdf]

DEGs	logFC	P.Value	sig	DEGs	logFC	P.Value	sig	DEGs	logFC	P.Value	sig
HSD11B1	4.08	<0.001	Up	TMEM255A	2.04	<0.001	Up	PIP	1.71	<0.001	Up
SCGB2A1	3.96	<0.001	Up	CDR1	2.02	<0.001	Up	INHBB	1.71	<0.001	Up
WIF1	3.94	<0.001	Up	ZDHHC11	2.02	<0.001	Up	CRY2	1.71	<0.001	Up
ZBTB16	3.48	<0.001	Up	R0SE4	2.02	<0.001	Up	SLC27A2	1.70	<0.001	Up
PM20D1	3.46	<0.001	Up	GPC3	2.01	<0.001	Up	44256.00	1.70	<0.001	Up
HMGCS2	3.45	<0.001	Up	SGCG	2.00	<0.001	Up	ZNF652	1.70	<0.001	Up
MSMB	3.28	<0.001	Up	MFSD4	2.00	<0.001	Up	CDO1	1.70	<0.001	Up
BTC	3.17	<0.001	Up	ZNF254	2.00	<0.001	Up	GPIHBP1	1.69	<0.001	Up
HAO2	3.08	<0.001	Up	C7	2.00	<0.001	Up	LOC285419	1.69	<0.001	Up
BPY2	3.07	<0.001	Up	PLA2G16	1.99	<0.001	Up	NR2F2-AS1	1.68	<0.001	Up
C14orf64	3.06	<0.001	Up	LOC100131541	1.99	<0.001	Up	PAK3	1.67	<0.001	Up
PHYHIP	3.01	<0.001	Up	GALNT15	1.98	<0.001	Up	AQP5	1.67	<0.001	Up
TMEM56	2.99	<0.001	Up	ADIPOQ	1.97	<0.001	Up	PRKCB	1.67	<0.001	Up
ELOVL3	2.93	<0.001	Up	FILIP1	1.97	<0.001	Up	VIT	1.67	<0.001	Up
WFDC3	2.89	<0.001	Up	PRR15L	1.97	<0.001	Up	BHLHE41	1.66	<0.001	Up
PAMR1	2.89	<0.001	Up	HS3ST6	1.97	<0.001	Up	MYOCD	1.66	<0.001	Up
FADS2	2.86	<0.001	Up	ACOX2	1.97	<0.001	Up	OSR1	1.66	<0.001	Up
BTBD16	2.85	<0.001	Up	CFTR	1.96	<0.001	Up	DACT1	1.65	<0.001	Up
FGFBP2	2.82	<0.001	Up	RERGL	1.96	<0.001	Up	SOAT1	1.64	<0.001	Up
ENPP5	2.80	<0.001	Up	TCF7L2	1.96	<0.001	Up	CLIC5	1.64	<0.001	Up
HIF3A	2.74	<0.001	Up	ADH1B	1.96	<0.001	Up	HPGDS	1.63	<0.001	Up
HSD3B1	2.74	<0.001	Up	TMEM116	1.95	<0.001	Up	FERMT2	1.63	<0.001	Up
GSTA3	2.74	<0.001	Up	SLC26A2	1.95	<0.001	Up	GABPB1-AS1	1.63	<0.001	Up
CCL27	2.72	<0.001	Up	LMOD1	1.94	<0.001	Up	KCNK5	1.62	<0.001	Up
LOC284578	2.70	<0.001	Up	MRAP	1.94	<0.001	Up	CYP2J2	1.62	<0.001	Up
CRAT	2.70	<0.001	Up	SOX8	1.93	<0.001	Up	PCDHB16	1.62	<0.001	Up
TNMD	2.70	<0.001	Up	LDB3	1.92	<0.001	Up	PRELP	1.62	<0.001	Up
KRT18	2.64	<0.001	Up	ADAMTS9-AS2	1.92	<0.001	Up	SLC25A18	1.62	<0.001	Up
THRSP	2.62	<0.001	Up	EPDR1	1.91	<0.001	Up	ZNF540	1.61	<0.001	Up
FABP7	2.60	<0.001	Up	GPAM	1.90	<0.001	Up	GATA6	1.61	<0.001	Up
APOC1	2.59	<0.001	Up	MGST1	1.89	<0.001	Up	PECR	1.61	<0.001	Up
KRT19	2.57	<0.001	Up	PCP4	1.88	<0.001	Up	IGFBP5	1.61	<0.001	Up
CLDN8	2.57	<0.001	Up	PLLP	1.88	<0.001	Up	C5orf46	1.61	<0.001	Up
LOC338667	2.55	<0.001	Up	SLC6A16	1.88	<0.001	Up	ACADL	1.61	<0.001	Up
BCAR3	2.52	<0.001	Up	EGF	1.87	<0.001	Up	P2RX1	1.61	<0.001	Up
LPL	2.51	<0.001	Up	DDIT4L	1.87	<0.001	Up	LINC00663	1.60	<0.001	Up
PLIN1	2.49	<0.001	Up	SCGB1D2	1.87	<0.001	Up	CORO2B	1.60	<0.001	Up
GLDC	2.48	<0.001	Up	FCHSD2	1.86	<0.001	Up	PROL1	1.60	<0.001	Up
ROPN1B	2.47	<0.001	Up	LRRN1	1.86	<0.001	Up	IGSF11	1.60	<0.001	Up
C1orf95	2.44	<0.001	Up	TMPRSS11E	1.86	<0.001	Up	TLN2	1.60	<0.001	Up
RBP4	2.44	<0.001	Up	CIDEC	1.86	<0.001	Up	GRB14	1.59	<0.001	Up
MYEOV	2.43	<0.001	Up	SDC2	1.86	<0.001	Up	GYPC	1.59	<0.001	Up
NR3C2	2.42	<0.001	Up	CASQ2	1.85	<0.001	Up	FIBIN	1.59	<0.001	Up
LEP	2.41	<0.001	Up	GSTM5	1.85	<0.001	Up	HRASLS5	1.59	<0.001	Up
SLC14A1	2.39	<0.001	Up	ADCY2	1.84	<0.001	Up	MIA	1.58	<0.001	Up
PPP1R1A	2.38	<0.001	Up	EMC3	1.84	<0.001	Up	PLEKHG5	1.58	<0.001	Up
CRISPLD1	2.37	<0.001	Up	TGFBR3	1.83	<0.001	Up	PAPLN	1.58	<0.001	Up
PLIN4	2.37	<0.001	Up	CHPT1	1.83	<0.001	Up	FST	1.57	<0.001	Up
ROPN1	2.37	<0.001	Up	PDE4DIP	1.83	<0.001	Up	PHYHD1	1.57	<0.001	Up

LPHN3	2.37	<0.001	Up	YBX2	1.82	<0.001	Up	TRIM2	1.57	<0.001	Up
PPP1R1B	2.33	<0.001	Up	PLN	1.82	<0.001	Up	FXYD6	1.56	<0.001	Up
IL37	2.31	<0.001	Up	LONRF1	1.82	<0.001	Up	ACTC1	1.56	<0.001	Up
KIAA1244	2.29	<0.001	Up	FAM189A2	1.81	<0.001	Up	CA3	1.56	<0.001	Up
TMEM132C	2.27	<0.001	Up	GXYLT2	1.80	<0.001	Up	PDZK1	1.56	<0.001	Up
AGR3	2.26	<0.001	Up	COPG2IT1	1.80	<0.001	Up	CCDC7	1.56	<0.001	Up
SCIN	2.26	<0.001	Up	MMP28	1.80	<0.001	Up	TAGLN	1.55	<0.001	Up
LOC100653086	2.23	<0.001	Up	TIMP3	1.79	<0.001	Up	LRRC17	1.55	<0.001	Up
TMEM139	2.23	<0.001	Up	C2orf40	1.79	<0.001	Up	PLCB4	1.54	<0.001	Up
MPZ	2.23	<0.001	Up	GAL	1.78	<0.001	Up	UPB1	1.54	<0.001	Up
ATP6V0A4	2.22	<0.001	Up	LOC157562	1.78	<0.001	Up	LAMB4	1.54	<0.001	Up
ESRRG	2.22	<0.001	Up	RHPN2	1.78	<0.001	Up	HYMAI	1.54	<0.001	Up
SEMA3E	2.21	<0.001	Up	ANG	1.77	<0.001	Up	FHL1	1.54	<0.001	Up
ANKRD33B	2.21	<0.001	Up	JADE1	1.77	<0.001	Up	LARP6	1.53	<0.001	Up
TNNI2	2.21	<0.001	Up	MCOLN3	1.77	<0.001	Up	DNER	1.53	<0.001	Up
HOTS	2.19	<0.001	Up	CPE	1.76	<0.001	Up	PCOLCE2	1.53	<0.001	Up
AWAT1	2.18	<0.001	Up	AGTR1	1.76	<0.001	Up	TACC1	1.53	<0.001	Up
KIAA1324	2.18	<0.001	Up	PDE9A	1.76	<0.001	Up	AK021804	1.53	<0.001	Up
MUC1	2.17	<0.001	Up	PPARG	1.75	<0.001	Up	PPP1R3C	1.52	<0.001	Up
KRT4	2.14	<0.001	Up	FOLR1	1.74	<0.001	Up	CHL1	1.52	<0.001	Up
SP8	2.13	<0.001	Up	EPCAM	1.74	<0.001	Up	RTN4	1.52	<0.001	Up
MIR143HG	2.13	<0.001	Up	COL28A1	1.73	<0.001	Up	LRRC37A3	1.52	<0.001	Up
TMEM47	2.12	<0.001	Up	HSPB6	1.73	<0.001	Up	LSP1	1.52	<0.001	Up
KRT77	2.11	<0.001	Up	PLP1	1.73	<0.001	Up	LONRF2	1.51	<0.001	Up
FA2H	2.11	<0.001	Up	RP11-757F18.5	1.73	<0.001	Up	MYBPC1	1.51	<0.001	Up
CYP4F8	2.10	<0.001	Up	TIMP4	1.73	<0.001	Up	DCAF16	1.51	<0.001	Up
TSPAN8	2.10	<0.001	Up	ACSBG1	1.73	<0.001	Up	LINC00312	1.51	<0.001	Up
MYOC	2.10	<0.001	Up	IGFBP6	1.73	<0.001	Up	MPPED2	1.51	<0.001	Up
C9orf152	2.08	<0.001	Up	CSPG4	1.73	<0.001	Up	DZIP1L	1.51	<0.001	Up
ZNF493	2.08	<0.001	Up	MB	1.72	<0.001	Up	STK32B	1.51	<0.001	Up
KRT79	2.06	<0.001	Up	RAI2	1.72	<0.001	Up	GS1-259H13.2	1.50	<0.001	Up
RNF150	2.06	<0.001	Up	RHOBTB3	1.71	<0.001	Up	FAM185A	1.50	<0.001	Up
AQP9	2.04	<0.001	Up	SSBP2	1.71	<0.001	Up	LOC101928784	1.50	<0.001	Up
FREM2	2.04	<0.001	Up	SYT8	1.71	<0.001	Up				

DEGs	logFC	P.Value	sig	DEGs	logFC	P.Value	sig	DEGs	logFC	P.Value	sig
S100A12	-9.69	<0.001	Down	TNIP3	-4.96	<0.001	Down	STS	-1.83	<0.001	Down
TMPRSS11D	-8.14	<0.001	Down	ALOX12B	-2.27	<0.001	Down	ACP5	-1.64	<0.001	Down
KYNU	-6.64	<0.001	Down	ISG20	-2.82	<0.001	Down	DESI1	-1.74	<0.001	Down
PLA2G4D	-5.86	<0.001	Down	KIF18B	-1.80	<0.001	Down	SPAG5	-1.54	<0.001	Down
CTA-384D8.35	-6.15	<0.001	Down	CENPW	-1.51	<0.001	Down	RAET1E	-2.03	<0.001	Down
SERPINB4	-9.53	<0.001	Down	CHI3L2	-3.57	<0.001	Down	IFI44	-2.14	<0.001	Down
TCN1	-7.94	<0.001	Down	RSAD2	-4.72	<0.001	Down	TRIM14	-2.06	<0.001	Down
SPRR2C	-7.46	<0.001	Down	SCO2	-2.80	<0.001	Down	DSC2	-2.60	<0.001	Down

PRSS27	-5.38	<0.001	Down	NDC80	-2.70	<0.001	Down	SDR9C7	-3.52	<0.001	Down
OASL	-7.15	<0.001	Down	LOC100996579	-2.25	<0.001	Down	PCDH7	-1.68	<0.001	Down
GDA	-6.76	<0.001	Down	GGH	-1.66	<0.001	Down	CXCL10	-3.89	<0.001	Down
KLK13	-5.93	<0.001	Down	PRSS3	-2.28	<0.001	Down	ZCCHC10	-2.12	<0.001	Down
TGM1	-4.69	<0.001	Down	LMNB2	-2.11	<0.001	Down	TMC5	-1.52	<0.001	Down
FUT3	-4.14	<0.001	Down	LYPD5	-3.38	<0.001	Down	UCA1	-2.48	<0.001	Down
SPTLC2	-3.91	<0.001	Down	PGM2	-1.85	<0.001	Down	NCAPG	-1.51	<0.001	Down
HYAL4	-4.79	<0.001	Down	TRIM62	-1.63	<0.001	Down	ID1	-2.18	<0.001	Down
CD24	-2.58	<0.001	Down	EIF4EBP1	-1.67	<0.001	Down	DTL	-1.84	<0.001	Down
ARSF	-4.62	<0.001	Down	SPRR2B	-3.36	<0.001	Down	PLA2G3	-1.84	<0.001	Down
ATP12A	-6.76	<0.001	Down	A2ML1	-2.57	<0.001	Down	XPO4	-1.74	<0.001	Down
GJB2	-1.76	<0.001	Down	XAF1	-2.05	<0.001	Down	PLCD4	-2.25	<0.001	Down
GZMB	-5.36	<0.001	Down	RGS1	-3.47	<0.001	Down	EHF	-2.44	<0.001	Down
KRT16	-2.98	<0.001	Down	RP11-164P12.3	-2.70	<0.001	Down	PTP4A1	-2.41	<0.001	Down
VNN1	-4.89	<0.001	Down	MAPK13	-1.50	<0.001	Down	MAPKAPK3	-1.99	<0.001	Down
FOXE1	-4.93	<0.001	Down	CDKN3	-2.94	<0.001	Down	UBE2T	-1.64	<0.001	Down
FAM110C	-1.65	<0.001	Down	KIF2C	-1.74	<0.001	Down	LTF	-3.64	<0.001	Down
CRABP2	-1.69	<0.001	Down	HN1	-1.59	<0.001	Down	LOC100288860	-1.73	<0.001	Down
RHCG	-5.98	<0.001	Down	RBBP6	-1.72	<0.001	Down	ICOS	-1.82	<0.001	Down
EPHB2	-4.21	<0.001	Down	CXCL13	-4.04	<0.001	Down	ECT2	-1.63	<0.001	Down
GBAP1	-1.66	<0.001	Down	FBXO6	-2.11	<0.001	Down	NDRG4	-1.61	<0.001	Down
SAMD9	-4.52	<0.001	Down	HAL	-1.56	<0.001	Down	CYB5R4	-1.59	<0.001	Down
PRKCQ	-4.91	<0.001	Down	ISG15	-3.49	<0.001	Down	PNPT1	-1.91	<0.001	Down
I0	-4.48	<0.001	Down	CDCA5	-2.80	<0.001	Down	CST7	-2.38	<0.001	Down
AKR1B10	-6.04	<0.001	Down	F12	-1.80	<0.001	Down	GYS1	-1.68	<0.001	Down
SERPINB3	-5.49	<0.001	Down	RABGGTA	-1.97	<0.001	Down	SPRR1A	-2.88	<0.001	Down
ARNTL2	-4.50	<0.001	Down	KLK6	-5.32	<0.001	Down	FAM26F	-2.54	<0.001	Down
HPSE	-5.55	<0.001	Down	CKS2	-1.55	<0.001	Down	C12orf75	-1.78	<0.001	Down
IL36G	-5.26	<0.001	Down	ASF1B	-2.15	<0.001	Down	USP18	-1.97	<0.001	Down
UPP1	-3.26	<0.001	Down	SLC2A1	-2.96	<0.001	Down	NRBF2	-1.63	<0.001	Down
SERPINB13	-2.42	<0.001	Down	GBP1	-2.78	<0.001	Down	MFHAS1	-1.72	<0.001	Down
TLE3	-3.13	<0.001	Down	HBEGF	-2.62	<0.001	Down	DENND1A	-2.09	<0.001	Down
POLR3G	-1.85	<0.001	Down	AIM2	-3.36	<0.001	Down	STX19	-1.52	<0.001	Down

CLEC7A	-4.13	<0.001	Down	AF086184	-3.08	<0.001	Down	APOBEC3B	-2.05	<0.001	Down
WNT5A	-2.54	<0.001	Down	CDCA2	-2.49	<0.001	Down	CD83	-1.90	<0.001	Down
CXCR2	-3.16	<0.001	Down	AASS	-1.68	<0.001	Down	IVL	-2.28	<0.001	Down
IGFL1	-5.26	<0.001	Down	EIF5	-1.71	<0.001	Down	UNC93A	-1.68	<0.001	Down
AMD1	-1.89	<0.001	Down	CKAP2L	-2.18	<0.001	Down	SBNO2	-1.58	<0.001	Down
PLBD1	-1.73	<0.001	Down	C12orf5	-2.63	<0.001	Down	PXMP4	-1.62	<0.001	Down
C12orf56	-3.56	<0.001	Down	SYNCRIP	-2.60	<0.001	Down	MMP12	-3.64	<0.001	Down
CCNE1	-2.36	<0.001	Down	NEK2	-1.94	<0.001	Down	LOC101928487	-1.59	<0.001	Down
MYO5A	-1.77	<0.001	Down	FUT1	-1.61	<0.001	Down	HEATR3	-1.57	<0.001	Down
LRP8	-3.26	<0.001	Down	LAMP3	-1.63	<0.001	Down	PRDM1	-1.61	<0.001	Down
MPZL2	-3.63	<0.001	Down	FGFBP1	-1.63	<0.001	Down	DHRS9	-2.95	<0.001	Down
CXCL8	-6.33	<0.001	Down	BIRC5	-3.11	<0.001	Down	CRCT1	-2.63	<0.001	Down
FCHSD1	-4.62	<0.001	Down	FAM65C	-2.56	<0.001	Down	IL26	-2.15	<0.001	Down
TTC39A	-2.79	<0.001	Down	CDCA8	-2.07	<0.001	Down	IL12RB1	-2.14	<0.001	Down
RAB27A	-4.15	<0.001	Down	DIO2	-3.36	<0.001	Down	TNFRSF21	-1.54	<0.001	Down
CDK5R1	-2.07	<0.001	Down	LRRC20	-1.65	<0.001	Down	CXCR6	-1.60	<0.001	Down
CCL20	-5.97	<0.001	Down	CYP4Z2P	-4.69	<0.001	Down	IFIT3	-1.73	<0.001	Down
ESYT3	-2.45	<0.001	Down	NUF2	-1.88	<0.001	Down	STEAP1B	-2.36	<0.001	Down
RDH16	-3.03	<0.001	Down	IFI6	-3.12	<0.001	Down	CXCL9	-3.44	<0.001	Down
MX1	-2.84	<0.001	Down	SERPI01	-3.62	<0.001	Down	CYP4F22	-2.12	<0.001	Down
S100A7A	-6.02	<0.001	Down	CDC45	-1.54	<0.001	Down	CH25H	-1.91	<0.001	Down
AK097453	-1.83	<0.001	Down	MOXD1	-2.00	<0.001	Down	RP1-93H18.6	-2.56	<0.001	Down
SLC26A9	-4.30	<0.001	Down	CYSRT1	-2.13	<0.001	Down	CXCL17	-2.35	<0.001	Down
CCNB1	-2.44	<0.001	Down	CTD-2619J13.13	-2.16	<0.001	Down	RP11-210K20.4	-1.51	<0.001	Down
EPN3	-2.37	<0.001	Down	CKAP4	-1.52	<0.001	Down	SULT2B1	-1.82	<0.001	Down
CHR09	-4.66	<0.001	Down	PLCD1	-1.65	<0.001	Down	E2F8	-1.54	<0.001	Down
DLGAP5	-3.17	<0.001	Down	DDX58	-1.61	<0.001	Down	XKRX	-1.99	<0.001	Down
PNP	-2.09	<0.001	Down	SLC7A5	-1.74	<0.001	Down	RQCD1	-1.62	<0.001	Down
CC02	-3.60	<0.001	Down	STEAP4	-2.70	<0.001	Down	MX2	-1.98	<0.001	Down
CXCL1	-5.89	<0.001	Down	IL19	-4.22	<0.001	Down	LINC00592	-2.43	<0.001	Down
N4BP1	-2.27	<0.001	Down	CFB	-1.67	<0.001	Down	ESPL1	-1.53	<0.001	Down

S100A9	-5.38	<0.001	Down	IL4R	-2.13	<0.001	Down	C15orf48	-2.15	<0.001	Down
DUOXA2	-2.28	<0.001	Down	KIF23	-2.04	<0.001	Down	BTBD19	-2.01	<0.001	Down
RRM2	-1.78	<0.001	Down	MKI67	-2.55	<0.001	Down	MIAT	-1.89	<0.001	Down
PI3	-3.50	<0.001	Down	ZNF165	-2.80	<0.001	Down	SAMSN1	-1.51	<0.001	Down
LINC01094	-3.11	<0.001	Down	SMC2	-1.92	<0.001	Down	CD3D	-2.29	<0.001	Down
SLC6A14	-2.38	<0.001	Down	TM9SF4	-1.94	<0.001	Down	MID2	-2.04	<0.001	Down
PPIF	-1.68	<0.001	Down	MPHOSPH6	-1.96	<0.001	Down	MAP2	-1.58	<0.001	Down
HELZ2	-3.00	<0.001	Down	CMPK2	-2.49	<0.001	Down	RASGEF1B	-1.69	<0.001	Down
CHAC1	-4.82	<0.001	Down	UGGT1	-1.70	<0.001	Down	AGPAT9	-1.58	<0.001	Down
EPSTI1	-4.55	<0.001	Down	PLAT	-3.04	<0.001	Down	PHKA1	-1.62	<0.001	Down
PARP9	-1.69	<0.001	Down	LINC01215	-2.55	<0.001	Down	SLC28A3	-1.59	<0.001	Down
GDPD3	-2.11	<0.001	Down	SMC4	-2.01	<0.001	Down	RDH12	-1.53	<0.001	Down
PLAGL2	-3.29	<0.001	Down	BUB1B	-2.16	<0.001	Down	SLC39A6	-2.67	<0.001	Down
SFT2D2	-2.85	<0.001	Down	CHAC2	-2.14	<0.001	Down	YOD1	-1.83	<0.001	Down
PGLYRP4	-3.31	<0.001	Down	AURKA	-2.64	<0.001	Down	GZMA	-2.39	<0.001	Down
RP11-295G20.2	-1.94	<0.001	Down	TGM3	-2.76	<0.001	Down	SPIRE1	-1.70	<0.001	Down
MICALL1	-1.71	<0.001	Down	GK	-2.29	<0.001	Down	LYNX1	-2.07	<0.001	Down
PGBD5	-3.22	<0.001	Down	EXO1	-1.78	<0.001	Down	PCP4L1	-2.63	<0.001	Down
BCL2A1	-3.39	<0.001	Down	SLC16A6	-2.24	<0.001	Down	CCL22	-1.89	<0.001	Down
SERPINB1	-2.23	<0.001	Down	HDHD1	-2.15	<0.001	Down	LOC102723864	-2.08	<0.001	Down
ATP13A4	-1.86	<0.001	Down	LCE3D	-2.92	<0.001	Down	SAMD9L	-1.87	<0.001	Down
DEPDC1B	-3.04	<0.001	Down	RAB27B	-2.03	<0.001	Down	IFI44L	-2.35	<0.001	Down
0MPT	-4.02	<0.001	Down	LRG1	-3.60	<0.001	Down	GPR183	-1.56	<0.001	Down
NT5C3A	-1.81	<0.001	Down	NUP50-AS1	-1.71	<0.001	Down	GUF1	-1.62	<0.001	Down
OAS3	-3.22	<0.001	Down	TPRXL	-2.04	<0.001	Down	MTCL1	-1.67	<0.001	Down
ADAMDEC1	-4.66	<0.001	Down	ASPM	-2.32	<0.001	Down	IL12B	-2.47	<0.001	Down
OAS2	-3.98	<0.001	Down	POLE2	-1.73	<0.001	Down	IL36A	-2.51	<0.001	Down
LCN2	-5.18	<0.001	Down	PDZK1IP1	-2.10	<0.001	Down	TLR2	-1.63	<0.001	Down
CHEK1	-2.52	<0.001	Down	DCTD	-1.76	<0.001	Down	IL1B	-2.53	<0.001	Down
TEX101	-3.76	<0.001	Down	IFIT1	-2.49	<0.001	Down	GOSR2	-1.74	<0.001	Down
NOD2	-2.06	<0.001	Down	DPH3	-1.69	<0.001	Down	HMOX1	-1.62	<0.001	Down
CYP7B1	-2.14	<0.001	Down	SHROOM2	-2.11	<0.001	Down	SIRPG	-1.61	<0.001	Down
LOC100506411	-3.47	<0.001	Down	ULBP2	-1.98	<0.001	Down	SPRR3	-3.46	<0.001	Down

AUNIP	-2.13	<0.001	Down	PUSL1	-1.61	<0.001	Down	MMP1	-3.36	<0.001	Down
CYP24A1	-3.30	<0.001	Down	AVEN	-1.67	<0.001	Down	CYP27B1	-2.14	<0.001	Down
PSME3	-1.73	<0.001	Down	TMEM86A	-2.95	<0.001	Down	CD2	-1.86	<0.001	Down
TNFRSF4	-1.92	<0.001	Down	AEN	-1.79	<0.001	Down	DCAF8	-1.52	<0.001	Down
GLUL	-2.68	<0.001	Down	C21orf91	-1.94	<0.001	Down	P2RY1	-1.87	<0.001	Down
NLRX1	-2.13	<0.001	Down	STYK1	-2.28	<0.001	Down	SOST	-3.07	<0.001	Down
TMEM57	-1.59	<0.001	Down	GM2A	-3.23	<0.001	Down	SELL	-1.66	<0.001	Down
UBE2N	-1.64	<0.001	Down	CCRN4L	-1.81	<0.001	Down	SGPP2	-1.89	<0.001	Down
ADAP2	-2.99	<0.001	Down	PRC1	-1.75	<0.001	Down	PSG7	-1.96	<0.001	Down
TC2N	-1.84	<0.001	Down	POR	-1.80	<0.001	Down	EML6	-1.67	<0.001	Down
KIF20A	-2.96	<0.001	Down	WARS	-1.65	<0.001	Down	KCTD21	-1.71	<0.001	Down
GALNT6	-3.30	<0.001	Down	SERTAD1	-1.61	<0.001	Down	ZIC2	-1.76	<0.001	Down
PTTG1	-1.53	<0.001	Down	SLCO4A1	-1.64	<0.001	Down	RC3H2	-1.55	<0.001	Down
KLK10	-2.36	<0.001	Down	GLT1D1	-1.75	<0.001	Down	SUSD4	-2.04	<0.001	Down
MELK	-2.66	<0.001	Down	ACPP	-2.95	<0.001	Down	ZYG11A	-2.06	<0.001	Down
IL36RN	-2.79	<0.001	Down	TUBG1	-1.66	<0.001	Down	LRRC55	-1.79	<0.001	Down
CENPE	-3.30	<0.001	Down	FAM83A	-2.33	<0.001	Down	TP53RK	-1.53	<0.001	Down
RTP4	-3.59	<0.001	Down	GBP5	-1.65	<0.001	Down	CLCN3	-1.63	<0.001	Down
TTC9	-3.26	<0.001	Down	C19orf66	-1.73	<0.001	Down	CCNO	-1.56	<0.001	Down
BZW1	-1.67	<0.001	Down	ZBED2	-3.29	<0.001	Down	ABCD3	-1.89	<0.001	Down
KIAA0101	-2.33	<0.001	Down	OSBPL3	-1.64	<0.001	Down	CASP5	-2.16	<0.001	Down
KRT6B	-1.75	<0.001	Down	STARD4	-2.59	<0.001	Down	LOC101927972	-1.98	<0.001	Down
SH3PXD2A-AS1	-2.99	<0.001	Down	UBE2C	-1.71	<0.001	Down	SLC26A4	-1.73	<0.001	Down
SOX7	-2.58	<0.001	Down	AGO2	-1.53	<0.001	Down	FCGR3B	-2.22	<0.001	Down
KRT6A	-1.72	<0.001	Down	SPCS3	-2.28	<0.001	Down	TUBBP5	-1.79	<0.001	Down
TMEM45B	-2.91	<0.001	Down	MXD1	-2.57	<0.001	Down	CBLC	-1.63	<0.001	Down
C17orf96	-3.16	<0.001	Down	GMFB	-2.04	<0.001	Down	PRKCH	-1.53	<0.001	Down
PRSS3P2	-1.74	<0.001	Down	OIP5	-1.52	<0.001	Down	HCAR3	-1.67	<0.001	Down
TMPRSS4	-4.79	<0.001	Down	PRSS53	-1.91	<0.001	Down	DOCK9-AS2	-1.54	<0.001	Down
CCNB2	-2.36	<0.001	Down	SHC1	-1.65	<0.001	Down	GABRA4	-1.87	<0.001	Down
SLC5A1	-3.45	<0.001	Down	VSNL1	-1.52	<0.001	Down	OCLN	-1.52	<0.001	Down
CARD6	-2.55	<0.001	Down	DDIAS	-2.19	<0.001	Down	PTPN22	-1.71	<0.001	Down

TPX2	-2.17	<0.001	Down	CDH26	-2.71	<0.001	Down	SERPI03	-2.64	<0.001	Down
APEX2	-1.90	<0.001	Down	ARG1	-2.13	<0.001	Down	GRHL1	-1.84	<0.001	Down
SPC25	-2.27	<0.001	Down	PKP1	-3.08	<0.001	Down	SLC39A2	-1.75	<0.001	Down
GK3P	-2.76	<0.001	Down	ZNF557	-1.86	<0.001	Down	SLAMF7	-1.55	<0.001	Down
TRIP13	-2.40	<0.001	Down	MAP7	-2.42	<0.001	Down	LOC101928231	-1.94	<0.001	Down
TTK	-2.81	<0.001	Down	CNNM4	-1.55	<0.001	Down	AMDHD1	-1.60	<0.001	Down
C10orf99	-4.86	<0.001	Down	OVOL1	-2.89	<0.001	Down	CCL8	-1.69	<0.001	Down
HS3ST3A1	-2.54	<0.001	Down	HMMR	-2.19	<0.001	Down	MYO10	-1.96	<0.001	Down
GRHL3	-2.40	<0.001	Down	NDC1	-1.56	<0.001	Down	APELA	-2.16	<0.001	Down
RIT1	-2.83	<0.001	Down	LOC101928100	-2.20	<0.001	Down	P4HB	-1.54	<0.001	Down
FAM83D	-2.52	<0.001	Down	AC005838.2	-2.09	<0.001	Down	0V3	-1.61	<0.001	Down
OAS1	-2.39	<0.001	Down	ABHD17C	-1.57	<0.001	Down	NWD2	-1.72	<0.001	Down
KCNJ15	-3.84	<0.001	Down	SLC27A4	-2.11	<0.001	Down	CHST1	-1.78	<0.001	Down
CEP55	-1.90	<0.001	Down	KIF4A	-2.50	<0.001	Down	CYP2E1	-1.56	<0.001	Down
LINC01214	-2.85	<0.001	Down	REN	-3.19	<0.001	Down	DF05	-1.75	<0.001	Down
APOL1	-2.39	<0.001	Down	SRXN1	-1.67	<0.001	Down	NEB	-1.56	<0.001	Down
SMPD3	-2.07	<0.001	Down	VMP1	-1.74	<0.001	Down	WFDC12	-2.53	<0.001	Down
ABCA12	-2.66	<0.001	Down	ST6GAL0C1	-2.42	<0.001	Down	EREG	-1.80	<0.001	Down
ATP10B	-2.57	<0.001	Down	MALT1	-1.71	<0.001	Down	PSORS1C2	-2.24	<0.001	Down
GFOD2	-1.75	<0.001	Down	GPX2	-2.26	<0.001	Down	CLIC3	-1.65	<0.001	Down
RAB7B	-1.79	<0.001	Down	CENPN	-2.03	<0.001	Down	SERPINB7	-1.83	<0.001	Down
IRF7	-2.55	<0.001	Down	ATP11B	-2.02	<0.001	Down	CDC42EP5	-1.60	<0.001	Down
PBK	-2.50	<0.001	Down	S100A8	-1.59	<0.001	Down	NLRP2	-2.76	<0.001	Down
DSG3	-3.16	<0.001	Down	ZBED6CL	-1.69	<0.001	Down	KCTD4	-2.39	<0.001	Down
RASGRP1	-1.65	<0.001	Down	LIPG	-3.54	<0.001	Down	HSD17B2	-2.50	<0.001	Down
FOSL1	-3.55	<0.001	Down	KLRB1	-2.52	<0.001	Down	SELE	-1.94	<0.001	Down
PRSS2	-1.90	<0.001	Down	EPHX3	-2.02	<0.001	Down	ANXA9	-2.71	<0.001	Down
CTPS1	-1.52	<0.001	Down	RAD51AP1	-1.84	<0.001	Down	HTR3A	-1.74	<0.001	Down
CNFN	-2.99	<0.001	Down	IKBKE	-2.11	<0.001	Down	CARD18	-1.51	<0.001	Down
STX6	-2.15	<0.001	Down	MTFR2	-1.59	<0.001	Down	NES	-1.50	<0.001	Down
G015	-2.08	<0.001	Down	E2F7	-2.08	<0.001	Down	IDO1	-1.52	<0.001	Down
TBC1D12	-2.67	<0.001	Down	TMEM40	-1.63	<0.001	Down	CD8A	-1.70	<0.001	Down
0PG	-2.21	<0.001	Down	SPRR1B	-1.72	<0.001	Down	HMGCS1	-1.78	<0.001	Down

PANX1	-1.63	<0.001	Down	FBXO45	-2.02	<0.001	Down	LAIR2	-1.53	<0.001	Down
IFI27	-1.98	<0.001	Down	CYP2C18	-2.88	<0.001	Down	PRR9	-3.04	<0.001	Down
BUB1	-2.41	<0.001	Down	FOXM1	-2.00	<0.001	Down	PSG4	-1.96	<0.001	Down
NIPAL4	-1.57	<0.001	Down	C16orf72	-2.11	<0.001	Down	KLK8	-1.83	<0.001	Down
NETO2	-2.76	<0.001	Down	SLC37A2	-1.64	<0.001	Down	LYZ	-1.96	<0.001	Down
BLM	-2.49	<0.001	Down	ANGPTL4	-3.17	<0.001	Down	SLURP1	-1.88	<0.001	Down
ACAP2	-2.34	<0.001	Down	KBTBD8	-1.59	<0.001	Down	CYP4F2	-1.56	<0.001	Down
CCL18	-2.07	<0.001	Down								

Supplementary Table 2 Trends of DEGs in NL-LS
