# Supplementary material for: A network pharmacology and molecular docking investigation on the mechanisms of Shanyaotianhua decoction (STT) as a therapy for psoriasis
Source: Medicine (Baltimore). 2023 Aug 25;102(34):e34859. doi: 10.1097/MD.0000000000034859 (PMC10470816; doi:10.1097/MD.0000000000034859)
Supplement: Supplementary file 5 [file medi-102-e34859-s005.pdf]

	GM2A	MMP12	RBP4	REN	S100A9
Docking (binding energy) (kcal/mol)					
spinasterol	-9.3	-8.09	-7.71	-7.53	-8.43
diosgenin	-8.99	-9.2	-7.97	-8.73	-7.77
24_Methylcholest	-6.95	-8.24	-7.4	-7.02	-7.41
RMSD ligand (nm)					
Spinasterol	1.540 (29 - 29 atoms)	0.264 (24 - 24 atoms)	1.949 (28 - 28 atoms)	0.909 (24 - 24 atoms)	4.772 (28 - 28 atoms)
diosgenin	0.000 (30 - 30 atoms)	0.001 (30 - 30 atoms)	0.000 (30 - 30 atoms)	0.001 (30 - 30 atoms)	0.000 (30 - 30 atoms)
24_Methylcholest	0.123 (22 - 22 atoms)	0.000 (21 - 21 atoms)	0.001 (21 - 21 atoms)	0.186 (22 - 22 atoms)	0.001 (21 -21 atoms)
RMSF backbone(nm)					
spinasterol	0.4	0.36	0.87	0.19	0.32
diosgenin	0.13	0.45	0.15	0.41	0.12
24_Methylcholest	0.12	0.24	0.21	0.19	0.11

Supplementary Table 3 Binding energy between compound and protein
