# Supplementary material for: A network pharmacology and molecular docking investigation on the mechanisms of Shanyaotianhua decoction (STT) as a therapy for psoriasis
Source: Medicine (Baltimore). 2023 Aug 25;102(34):e34859. doi: 10.1097/MD.0000000000034859 (PMC10470816; doi:10.1097/MD.0000000000034859)

Supplementary Figure 3| Experimental design for elucidating the mechanism(s) of action of STT in the treatment of psoriasis.

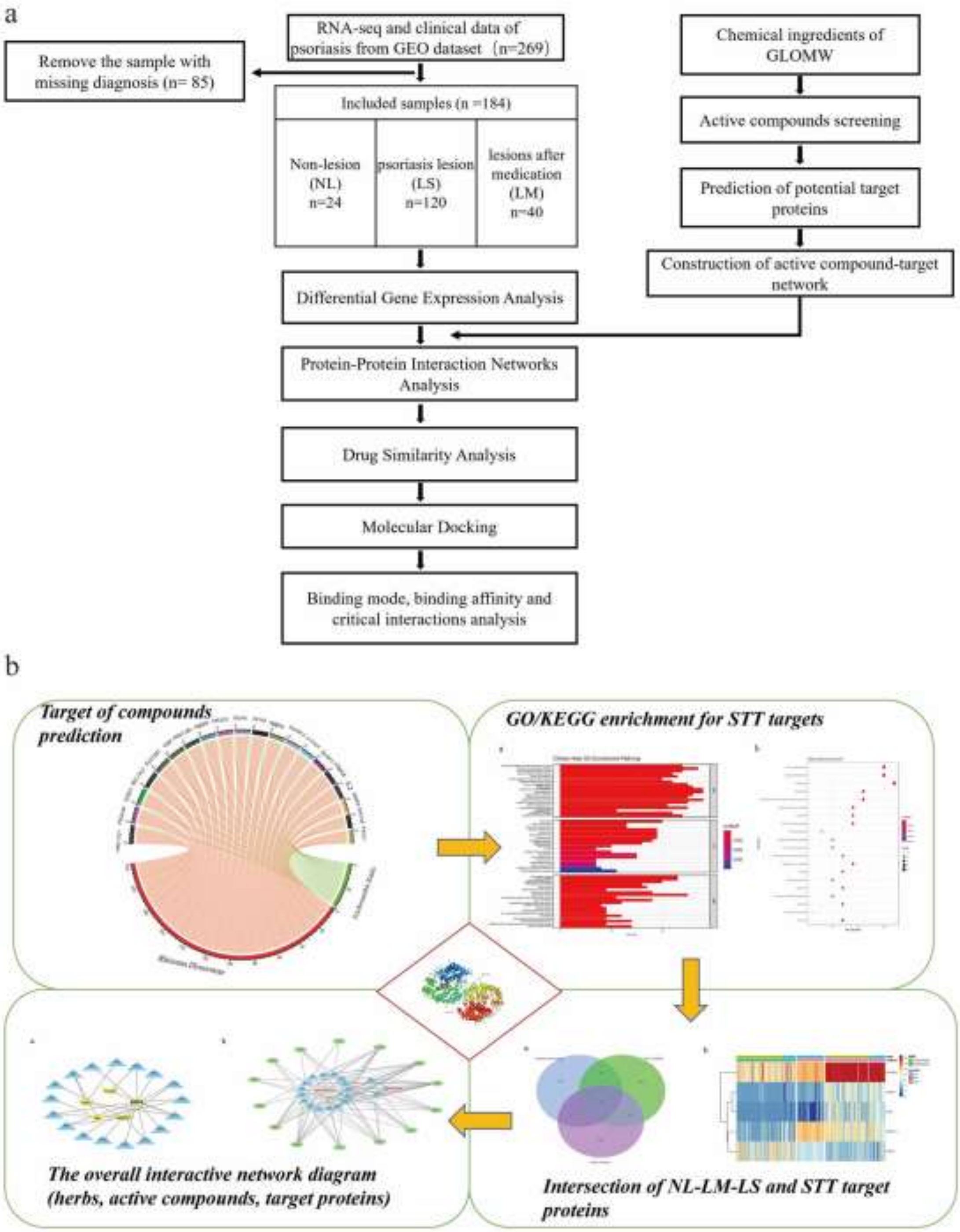

Supplement: Supplementary file 6 [file medi-102-e34859-s006.pdf]
